# Very low concentration of lipopolysaccharide can induce the production of various cytokines and chemokines in human primary monocytes

**DOI:** 10.1186/s13104-022-05941-4

**Published:** 2022-02-10

**Authors:** Ratthakorn Chaiwut, Watchara Kasinrerk

**Affiliations:** 1 Division of Clinical Immunology, Department of Medical Technology, Faculty of Associated Medical Sciences, Chiang Mai University, Chiang Mai, Thailand; 2 Biomedical Technology Research Center, National Center for Genetic Engineering and Biotechnology, National Science and Technology Development Agency at the Faculty of Associated Medical Sciences, Chiang Mai University, Chiang Mai, Thailand

**Keywords:** Chemokine, Innate immunity, Lipopolysaccharide, Monocyte, Proinflammatory cytokine

## Abstract

**Objective:**

Lipopolysaccharide (LPS), a component of gram-negative bacteria, is a potent innate immune stimulus. The interaction of LPS with innate immune cells induces the production of proinflammatory cytokines and chemokines, thereby leading to the control of infection. In the present study, we investigated the effect of a wide range of LPS concentrations on the regulation of various proinflammatory cytokines and chemokines in human primary monocytes and T lymphocytes.

**Results:**

We demonstrated that a very low concentration of LPS could regulate the production of cytokines and chemokines in monocytes but not T lymphocytes. Unexpectedly, very low concentrations of LPS (0.0025 and 0.005 ng/mL) could induce TNF-α and IL-6 production, respectively, in monocytes. Our findings provide evidence that in the presence of monocytes, even very low endotoxin contamination could induce cytokine production. We suggest that the recombinant proteins used to investigate immune functions must be thoroughly screened for endotoxins using a highly sensitive method.

**Supplementary Information:**

The online version contains supplementary material available at 10.1186/s13104-022-05941-4.

## Introduction

Lipopolysaccharide (LPS) is a component of the outer membrane of gram-negative bacteria. The interaction of LPS with innate immune cells, especially monocytes and macrophages, triggers inflammatory responses to control pathogen infection [1–3]. The activation of innate immune cells by LPS is initiated by the sequential actions of LPS-binding protein (LBP), CD14, MD-2, and TLR-4 [1, 4–7]. LBP, in concert with CD14, presents LPS molecules to the MD-2/TLR4 receptor complex, which leads to the activation of MyD88- and TRIF-dependent signalling pathways [1, 6, 8]. The MyD88-dependent signalling pathway facilitates the rapid activation of NF-κB, thereby leading to the production of various proinflammatory cytokines and chemokines. Conversely, TRIF-dependent pathways lead to the activation of the transcription factor IRF3 and promote the production of type I IFN and IFN-dependent chemokines. Therefore, activation of innate immune cells by LPS initiates cross-talk between innate and adaptive immune responses [1, 9, 10].

Currently, many recombinant proteins are used to investigate immune functions. Many of these proteins, which are generated in-house and are commercially available, are produced in *Escherichia coli* and may be contaminated by endotoxin. The amount of endotoxin contamination is usually stated by researchers or on the data sheets accompanying commercial products, and that the endotoxin level is less than 1 endotoxin unit (EU), which is equivalent to 0.1 ng of *E. coli* LPS per µg of protein [11]. However, concerns about monocytes, which are highly sensitive to LPS, have been raised [1, 2, 12]. Residual endotoxin contamination in the recombinant protein used might be sufficient to activate monocytes [1, 12] and lead to misinterpretation when using the recombinant protein to validate immune responses. In the present study, we assessed the effect of a wide range of LPS concentrations on the production of various proinflammatory cytokines and chemokines in human primary monocytes and T lymphocytes. The range of LPS concentrations and the cytokines/chemokines studied were different from those documented. We demonstrated that a very low concentration of LPS (0.0025 ng/mL) was able to activate monocytes. These results suggest the importance of awareness of LPS contamination in any substance when working with monocytes.

## Main text

### Methods

#### Antibodies, reagents, and cells

LPS from *Escherichia coli* serotype 055:B5 was purchased from Sigma-Aldrich (Taufkirchen, Germany). The anti-CD3 monoclonal antibody (mAb) clone OKT3 and anti-CD28 mAb clone L293 were purchased from Ortho Pharmaceuticals (Raritan, NJ, USA) and BD Bioscience (San Jose, CA, USA), respectively. FITC-labelled anti-CD3 mAbs were purchased from BD Biosciences (San Jose, CA, USA). PerCP-labelled anti-CD14 mAbs and PE-conjugated anti-IFN-γ, anti-TNF-α, anti-IL-6, and anti-IL-10 were purchased from BioLegend (San Diego, CA, USA). PE-conjugated anti-GM-CSF, anti-IL-1β, anti-CCL2, anti-CCL3, anti-CCL4, and anti-CXCL10 mAbs were purchased from Miltenyi Biotech (Bergisch Gladbach, Germany). Brefeldin A and monensin were purchased from BioLegend (San Diego, CA, USA).

Peripheral blood mononuclear cells (PBMCs) were isolated from healthy donors using Ficoll-Hypaque (IsoPrep; Robbins Scientific Corporation, Sunnyvale, CA, USA) gradient centrifugation.

#### LPS stimulation

PBMCs were seeded in a 24-well culture plate (Costar; Corning, NY, USA) at a concentration of 1 × 10^6^ cells per well. The cells were stimulated with various concentrations of LPS, immobilized anti-CD3 mAbs (25 ng/mL) and soluble anti-CD28 mAbs (50 ng/mL) as a T cell activation control or RPMI containing 10% FBS as an unstimulated control. Cells were incubated at 37 °C in a humidified incubator with 5% CO_2_. After 1 h of incubation, 1 μg/mL brefeldin A and 1 μM monensin were added and continuously incubated for 5 h. Cells were then harvested for further intracellular staining.

#### Intracellular cytokine staining

After being stimulated, the cells were washed twice with phosphate-buffered saline (PBS) containing 1% BSA and 0.1% NaN_3_. The cells were then fixed with 4% paraformaldehyde for 15 min at room temperature, washed and blocked with 10% heat-inactivated human blood group AB serum. The blocked cells were then stained with fluorochrome-conjugated anti-CD3, anti-CD14, anti-IFN-γ, anti-TNF-α, anti-GM-CSF, anti-IL-1β, anti-IL-6, anti-IL-10, anti-CCL2, anti-CCL3, anti-CCL4, and anti-CXCL10 mAbs or isotype-matched control mAbs for 30 min at 4 °C. The cells were then washed and fixed with PBS containing 1% paraformaldehyde. The stained cells were quantified using a flow cytometer (BD Accuri™ C6 flow cytometer; BD Biosciences) and analysed using FlowJo software. During analysis, monocytes and T lymphocytes were gated based on the expression of CD14 and CD3, respectively.

#### Statistical analysis

The data are expressed as the mean ± standard error of the mean (SEM). All statistical analyses were performed using GraphPad Prism software version 9.1.2 (GraphPad Software, CA, USA). One-way ANOVA with Tukey’s multiple comparison test was used to compare the means of each group.

### Results

#### Induction of cytokine and chemokine production in human primary monocytes by LPS

**Fig. 1:**
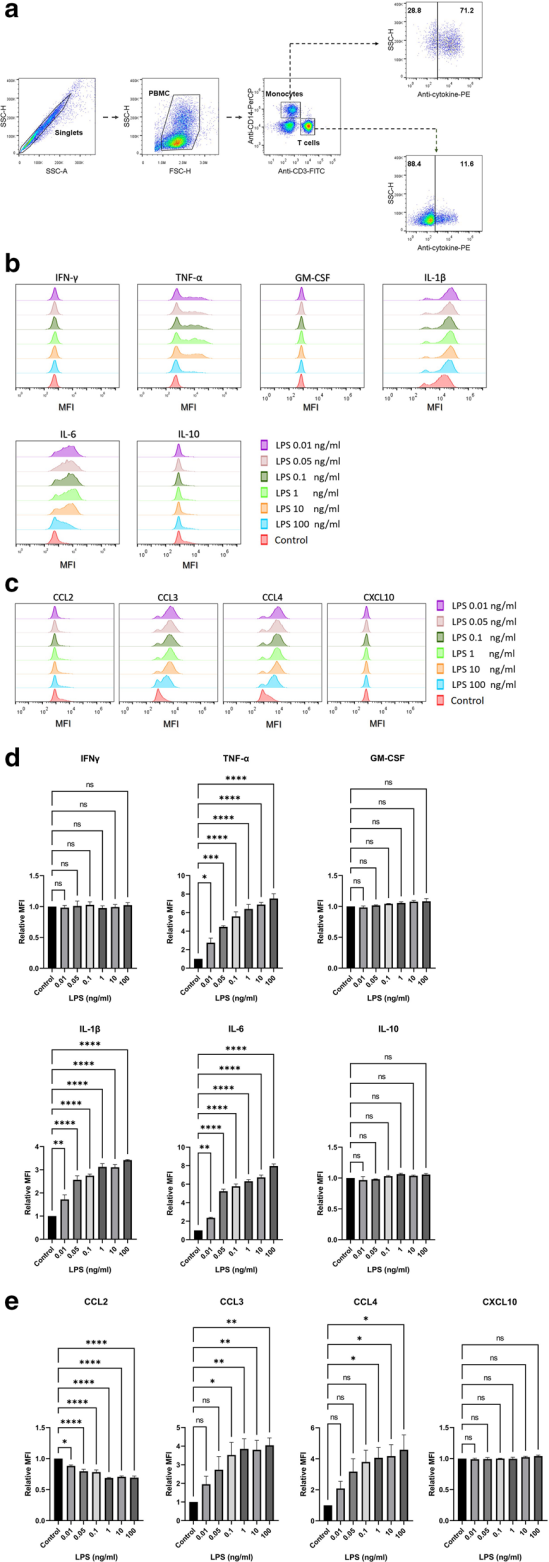
Lipopolysaccharide induces the production of various cytokines and chemokines in monocytes. PBMCs (n = 3) were stimulated with the indicated concentrations of LPS or in the absence of LPS (Control). The intracellular cytokines and chemokines were determined by flow cytometry. The gating strategies on CD14+ monocytes and CD3+ T lymphocytes are shown (**a**). The histogram plots from one of the three individuals on indicated cytokines (**b**) and chemokines (**c**) produced in CD14+ monocytes are indicated. The bar graphs indicated relative fluorescent intensity (MFI) of cytokines (**d**) or chemokines (**e**) are expressed as mean ± standard error of mean (SEM). Statistical analysis was performed using one-way ANOVA with Tukey’s multiple comparison test. *; *p* ≤ 0.05, **; *p* ≤ 0.01, ***; *p* ≤ 0.001, and ****; *p* ≤ 0.0001. *NS* not significant

To determine the effect of LPS on the activation of human primary monocytes and lymphocytes, we first activated PBMCs with LPS concentrations ranging from 0.01 to 100 ng/mL. The cells were then intracellularly stained for various cytokines (IFN-γ, TNF-α, GM-CSF, IL-1β, IL-6, and IL-10) and chemokines (CCL2, CCL3, CCL4, and CXCL10). Cytokine and chemokine production in monocytes and T lymphocytes was determined by flow cytometry. Gating strategies for monocytes and T lymphocytes were based on their signature surface molecules and are shown in Fig. 1a.

In monocytes, different cytokines and chemokines exhibited dissimilar responses to LPS stimulation (Fig. 1b, c). In comparison to the unstimulated control, all concentrations of LPS induced the production of proinflammatory cytokines, including TNF-α, IL-1β and IL-6, but not IL-10, GM-CSF, or IFN-γ (Fig. 1d). Among chemokines, the upregulation of CCL3 and CCL4 production was observed in response to 0.1 ng/mL LPS or higher and 1 ng/mL LPS or higher, respectively (Fig. 1e). No upregulation of CXCL10 expression was observed at any LPS concentration used (Fig. 1e). Conversely, CCL2 production was downregulated in response to all concentrations of LPS (Fig. 1e). The flow cytometric profiles of each subject and data analysis are shown in Additional file 1: Fig. S1 and Additional file 5: Table S1).

**Fig. 2:**
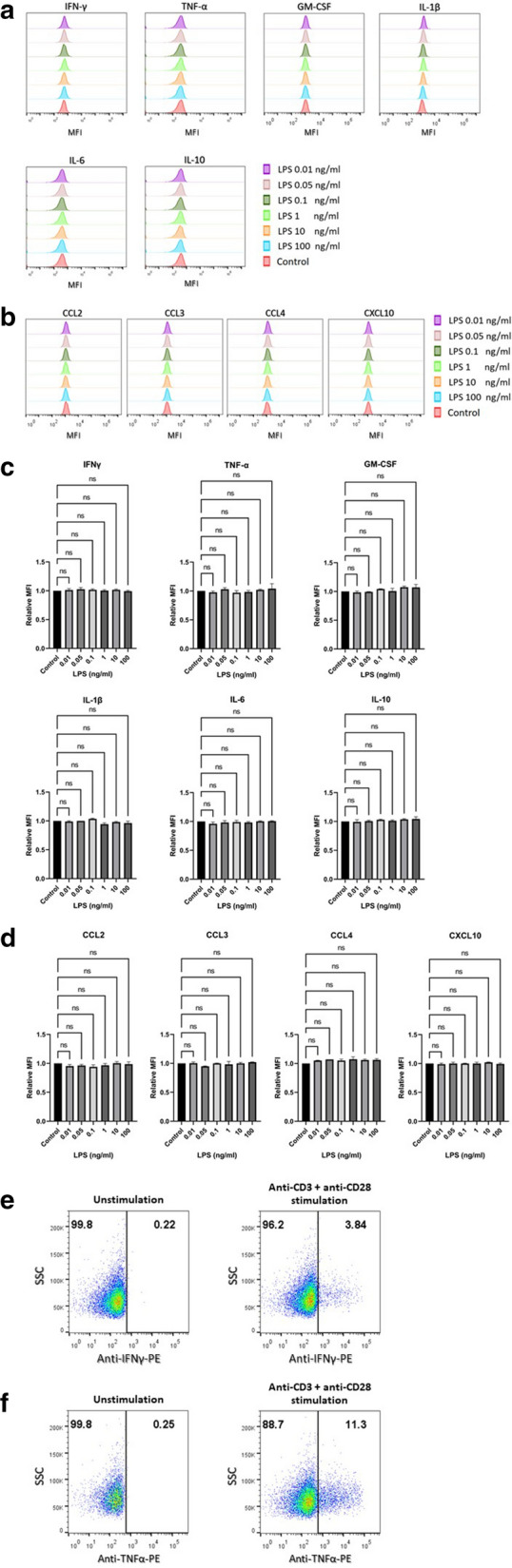
The production of cytokines and chemokines in T lymphocytes upon lipopolysaccharide activation. PBMCs (n = 3) were stimulated with the indicated concentrations of LPS or in the absence of LPS (Control). Intracellular cytokines and chemokines were determined using flow cytometry. CD3+ T lymphocyte population was gated and histogram plots from one of the three individuals on indicated cytokines (**a**) and chemokines (**b**) are shown. The bar graphs indicated relative mean fluorescent intensity (MFI) of cytokines (**c**) and chemokines (**d**) are expressed as mean ± standard error of mean (SEM). The dot plot showing the expression of IFN-γ (**e**) and TNF-α (**f**) upon stimulation with anti-CD3 and anti-CD28 mAbs or unstimulated control are shown. Statistical analysis was performed using one-way ANOVA with Tukey’s multiple comparison test. *NS* not significant

In T lymphocytes, no concentrations of LPS affected any tested cytokine or chemokine (Fig. 2a–d). Anti-CD3 and anti-CD28 mAbs were used as T lymphocyte activation control, and we found that IFN-γ and TNF-α were induced upon T cell activation (Fig. 2e, f). The flow cytometric profiles of each subject and data analysis are shown in Additional file 2: Fig. S2, Additional file 3: Fig. S3 and Additional file 6: Table S2).

#### Very low concentrations of LPS induce TNF-α and IL-6 production

**Fig. 3:**
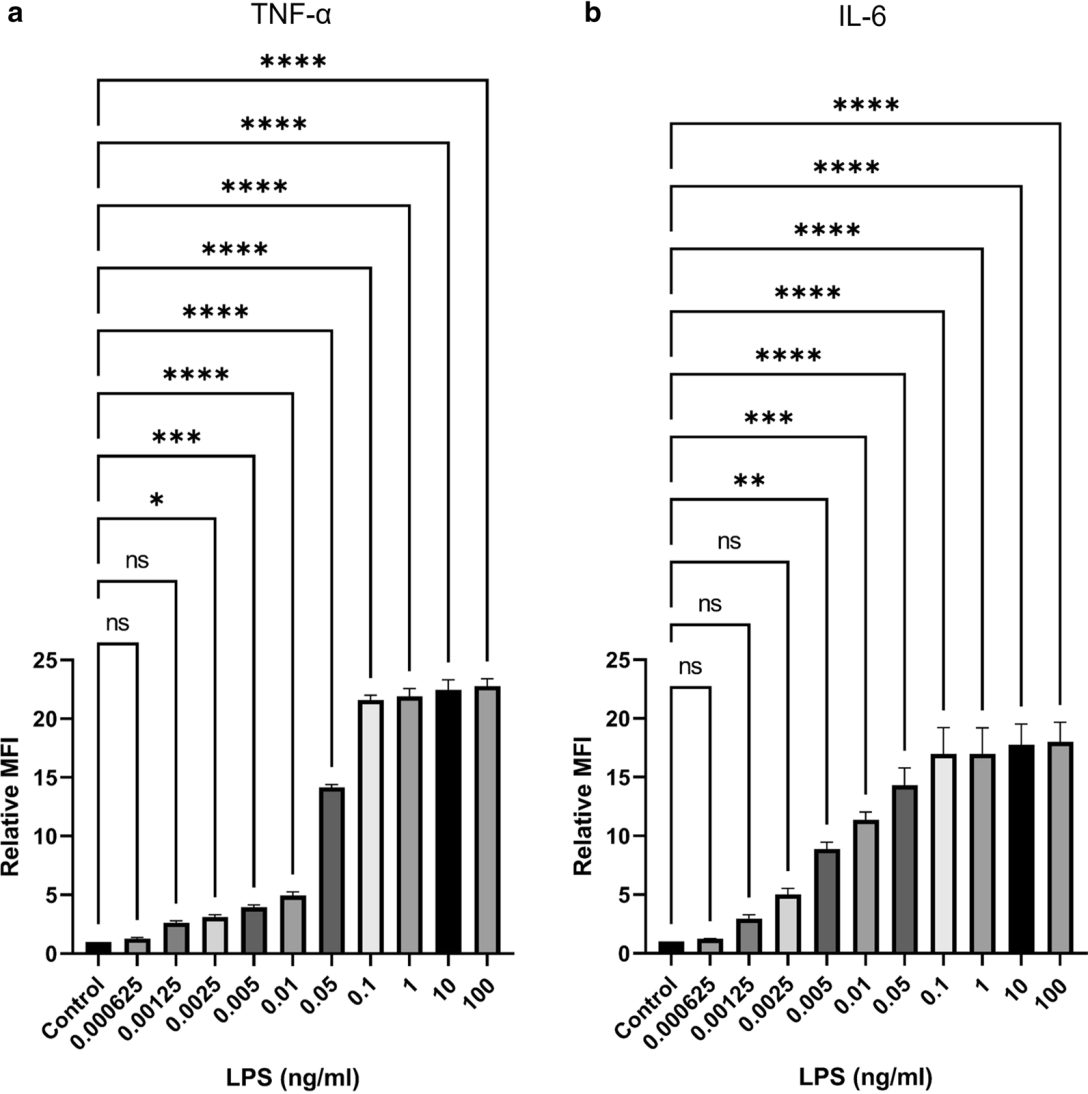
A very low concentrations of lipopolysaccharide induce the production of TNF-α and IL-6 in monocytes. PBMCs (n = 3) were stimulated with the indicated concentrations of LPS or in the absence of LPS (Control). CD14+ monocytes were gated and the bar graphs indicated the relative mean fluorescent intensity (MFI) of TNF-α (**a**) and IL-6 (**b**) are expressed as mean ± standard error of mean (SEM). Statistical analysis was performed using one-way ANOVA with Tukey’s multiple

As described above, a low concentration of LPS (0.01 ng/mL) induced the production of TNF-α and IL-6. We determined whether even lower concentrations of LPS (0.000625–100 ng/mL) could induce TNF-α and IL-6 production. Surprisingly, at lower concentrations, the effect of LPS on TNF-α and IL-6 production could still be observed (Fig. 3). Very low concentrations of LPS (0.0025 and 0.005 ng/mL) could induce TNF-α and IL-6 production, respectively (Fig. 3a, b). The flow cytometric profiles of each subject and data analysis are shown in Additional file 4: Fig. S4 and Additional file 7: Table S3).

As expected, T lymphocytes were not activated by LPS, whereas the anti-CD3 and anti-CD28 mAb control exhibited stimulatory effects (data not shown).

### Discussion

LPS is a glycan-based gram-negative pathogen-associated molecular pattern (PAMP) that induces innate immune responses in a sequential manner starting with the binding of LPS to LPS-binding protein, which is then transferred to CD14, and finally interacts with the TLR-4/MD-2 complex [6, 13–15]. Bacterial LPS has been demonstrated to exhibit potent immunostimulatory activity and induce the production of various proinflammatory mediators to control bacterial infection. While several studies have reported the effect of LPS on various cell types [1, 12], studies comparing whether various concentrations of LPS affect the production of cytokines and chemokines in human primary monocytes and T lymphocytes are limited. Because the activation of CD14+ monocytes by LPS leads to the production of cytokines and PBMCs consist of monocytes and lymphocytes, we examined whether while PBMC activation by LPS and the cytokines produced by activated monocytes might have an effect on CD3+ T lymphocytes and induce cytokine production. In the present study, PBMCs were activated with LPS, and cytokine and chemokine production by monocytes and T lymphocytes was examined.

We demonstrated that LPS could regulate the production of cytokines and chemokines in monocytes. However, LPS did not affect T lymphocytes. These results indicated that the cytokines and chemokines produced by LPS-activated monocytes have no influence on cytokine production by T lymphocytes. Surprisingly, we found that a very low concentration of LPS could induce TNF-α and IL-6 production in monocytes. TNF-α is the prototypical inflammatory cytokine and is involved in several cellular processes, including cytokine and acute phase protein production, adhesion molecule expression, cell proliferation, differentiation, and cell death [1]. IL-6 is also a proinflammatory cytokine. Conversely, IL-6 also plays an anti-inflammatory role [1, 16–18]. The different activities of IL-6 may depend on the type of signalling that is induced, either through membrane-bound or soluble IL-6R [1, 18]. TNF-α and IL-6 thus contribute to many pathophysiological processes, including endotoxic shock [2, 19]. Schwarz et al*.* demonstrated that 0.2 ng/mL LPS could induce the production of TNF-α and IL-6 by CD14+ monocytes [12]. Our findings, however, demonstrated that a much lower concentration (0.0025 ng/mL) of LPS could induce proinflammatory cytokines. The different observations may come from the method used. Purified monocytes were used in the previous study [12], while PBMCs were used in the present study. In addition, secreted cytokines were measured in the previous report [12], while intracellular cytokines were measured in the present study. Similar to the production of proinflammatory cytokines, LPS-induced production of chemokines in innate immune cells has also been reported [1, 20–22]. In agreement with previous reports, our study revealed that LPS is a potent stimulus that induces the production of chemokines. However, the effect of LPS on the induction of chemokines was lower than that observed for cytokines. CCL3 and CCL4 were upregulated in monocytes by 0.1 and 1 ng/mL LPS, respectively. However, LPS had no effect on CXCL10 production and reduced CCL2 production.

LPS stimulates monocytes via innate receptors, including CD14, and the TLR-4/MD-2 complex [1, 4–6, 23, 24]. However, in the absence of CD14, a high concentration of LPS can directly stimulate TLR4 and MD-2 [23, 25]. It was demonstrated that B cells express the receptors RP105 and MD-1, which are structurally related to TLR-4 and MD-2. These receptors are responsible for LPS signalling in B cells [26, 27]. However, the direct effect of LPS on T lymphocytes is largely unknown. Innate immune stimulation by LPS positively impacts T lymphocyte priming. LPS stimulation causes antigen presenting cells (APCs) to upregulate MHC class II molecules, costimulatory molecules, and cytokines that boost T lymphocyte function [23]. A number of publications, controversially, reported evidence for the activation of T lymphocytes by LPS and demonstrate that this activation requires direct cell-to-cell contact with monocytes and costimulatory signals provided by the B7 and CD28 interaction [28–31]. In the present study, however, we found that LPS had no effect on T lymphocytes, which is in line with previous reports [32–34]. The concentrations of LPS and the stimulation time used in our study, however, were different from the reports that showed the effect of LPS on T lymphocytes [28–31].

In summary, we demonstrated that very low concentrations of LPS can induce the production of various cytokines and chemokines in human primary monocytes. This information is important, as low amounts of endotoxin impurities can be found in recombinant proteins produced by *E. coli* [12] and might be sufficient to activate monocytes. These results, therefore, could generate erroneous data when using LPS-contaminated materials to investigate immune functions. To avoid endotoxin contamination, we recommend working with recombinant proteins that have been expressed under endotoxin-free conditions, such as in mammalian cells. In addition, removing LPS from the recombinant proteins using a very effective LPS elimination method is required to avoid the activation of LPS-sensitive immune cells.

## Limitations

We demonstrated that LPS have no effect on T lymphocytes. However, in our study, PBMCs were incubated with LPS for only 6 h. Additional investigations using longer incubation times of LPS and PBMCs are required to confirm that LPS has no effect on T lymphocytes among PBMCs.

## Supplementary Information

**Additional file 1: Figure S1.** Flow cytometric profiles of each subject (according to Fig. 1 in the paper): lipopolysaccharide induces the production of various cytokines and chemokines in monocytes. PBMCs were stimulated with the indicated concentrations of LPS or in the absence of LPS (Control). The intracellular cytokines and chemokines were determined by flow cytometry. CD14+ monocyte population of the three individuals (as indicated) were gated and dot plotted on the expression of the indicated cytokines and chemokines are shown (numbers indicate the % cells).

**Additional file 2: Figure S2.** Flow cytometric profiles of each subject (according to Fig. 2 in the paper): the production of cytokines and chemokines in T lymphocytes upon lipopolysaccharide activation. PBMCs (n = 3) were stimulated with the indicated concentrations of LPS or in the absence of LPS (Control). Intracellular cytokines and chemokines were determined using flow cytometry. CD3+ T lymphocyte population of the three individuals (as indicated) were gated and dot plotted on the expression of the indicated cytokines and chemokines are shown (numbers indicate the % cells).

**Additional file 3: Figure S3.** Flow cytometric profiles of each subject (according to Fig. 2 in the paper): the production of TNF-α and IFN-γ in T lymphocytes upon anti-CD3 and anti-CD28 mAbs activation. PBMCs were stimulated with anti-CD3 and anti-CD28 mAbs or or unstimulated control (as indicated). Intracellular cytokines were determined using flow cytometry. CD3+ T lymphocyte population of the three individuals (as indicated) were gated and dot plotted on the expression of the TNF-α and IFN-γ upon stimulation with anti-CD3 and anti-CD28 mAbs or unstimulated control are shown (numbers indicate the % cells).

**Additional file 4: Figure S4.** Flow cytometric profiles of each subject (according to Fig. 3 in the paper): lipopolysaccharide induces the production of TNF-α and IL-6 in monocytes. PBMCs were stimulated with the indicated concentrations of LPS or in the absence of LPS (Control). The intracellular TNF-α and IL-6 were determined by flow cytometry. CD14+ monocyte population of the three individuals (as indicated) were gated and dot plotted on the expression of the indicated cytokines are shown (numbers indicate the % cells).

**Additional file 5: Table S1.** Data analyzed form flow cytometric profiles of each subject (according to Fig. 1 in the paper): lipopolysaccharide induces the production of various cytokines and chemokines in monocytes. PBMCs were stimulated with the indicated concentrations of LPS. The intracellular cytokines and chemokines were determined by flow cytometry. CD14+ monocyte population of the three individuals (as indicated) were gated and mean fluorescence intensity of the expression of the indicated cytokines and chemokines are shown.

**Additional file 6: Table S2.** Data analyzed form flow cytometric profiles of each subject (according to Fig. 2 in the paper): lipopolysaccharide induces the production of various cytokines and chemokines in T lymphocytes. PBMCs were stimulated with the indicated concentrations of LPS. The intracellular cytokines and chemokines were determined by flow cytometry. CD3+ T lymphocyte population of the three individuals (as indicated) were gated and mean fluorescence intensity of the expression of the indicated cytokines and chemokines are shown.

**Additional file 7: Table S3.** Data analyzed form flow cytometric profiles of each subject (according to Fig. 3 in the paper): lipopolysaccharide induces the production of TNF-α and IL-6 in monocytes. PBMCs were stimulated with the indicated concentrations of LPS. The intracellular TNF-α and IL-6 were determined by flow cytometry. CD14+ monocyte population of the three individuals (as indicated) were gated and mean fluorescence intensity of the expression of the indicated cytokines are shown.

## Data Availability

All data generated or analyzed during the current study are available in Additional files.

## Abbreviations

BSA: Bovine serum albumin
CCL: Chemokine (C–C motif) ligand
CD: Cluster of differentiation
CXCL: Chemokine (C-X-C motif) ligand
FBS: *Fetal bovine serum*
FITC: Fluorescein isothiocyanate
FSC: Forward scatter
GM-CSF: Granulocyte–macrophage colony-stimulating factor
IFN: Interferon
IL: Interleukin
IL-6R: Interleukin-6 receptor
IRF3: Interferon regulatory transcription factor 3
MD-2: Myeloid differentiation 2
MHC: Major histocompatibility complex
MyD88: Myeloid differentiation primary response 88
NF-κB: Nuclear factor kappa light chain enhancer of activated B cells
PE: Phycoerythrin
PerCP: Peridinin Chlorophyll Protein Complex
RP105: Radioprotective 105 kDa
SSC: Side scatter
TLR: Toll-like receptor
TNF: Tumor necrosis factor
TRIF: TIR (Toll/interleukin-1 receptor) domain-containing adaptor protein inducing interferon beta
