# Supplementary material for: Very low concentration of lipopolysaccharide can induce the production of various cytokines and chemokines in human primary monocytes
Source: BMC Res Notes. 2022 Feb 10;15:42. doi: 10.1186/s13104-022-05941-4 (PMC8832778; doi:10.1186/s13104-022-05941-4)
Supplement: Supplementary file 1 — Additional file 1: Figure S1. Flow cytometric profiles of each subject (according to Fig. 1 in the paper): lipopolysaccharide induces the production of various cytokines and chemokines in monocytes. PBMCs were stimulated with the indicated concentrations of LPS or in the absence of LPS (Control). The intracellular cytokines and chemokines were determined by flow cytometry. CD14+ monocyte population of the three individuals (as indicated) were gated and dot plotted on the expression of the indicated cytokines and chemokines are shown (numbers indicate the % cells). [file 13104_2022_5941_MOESM1_ESM.docx]

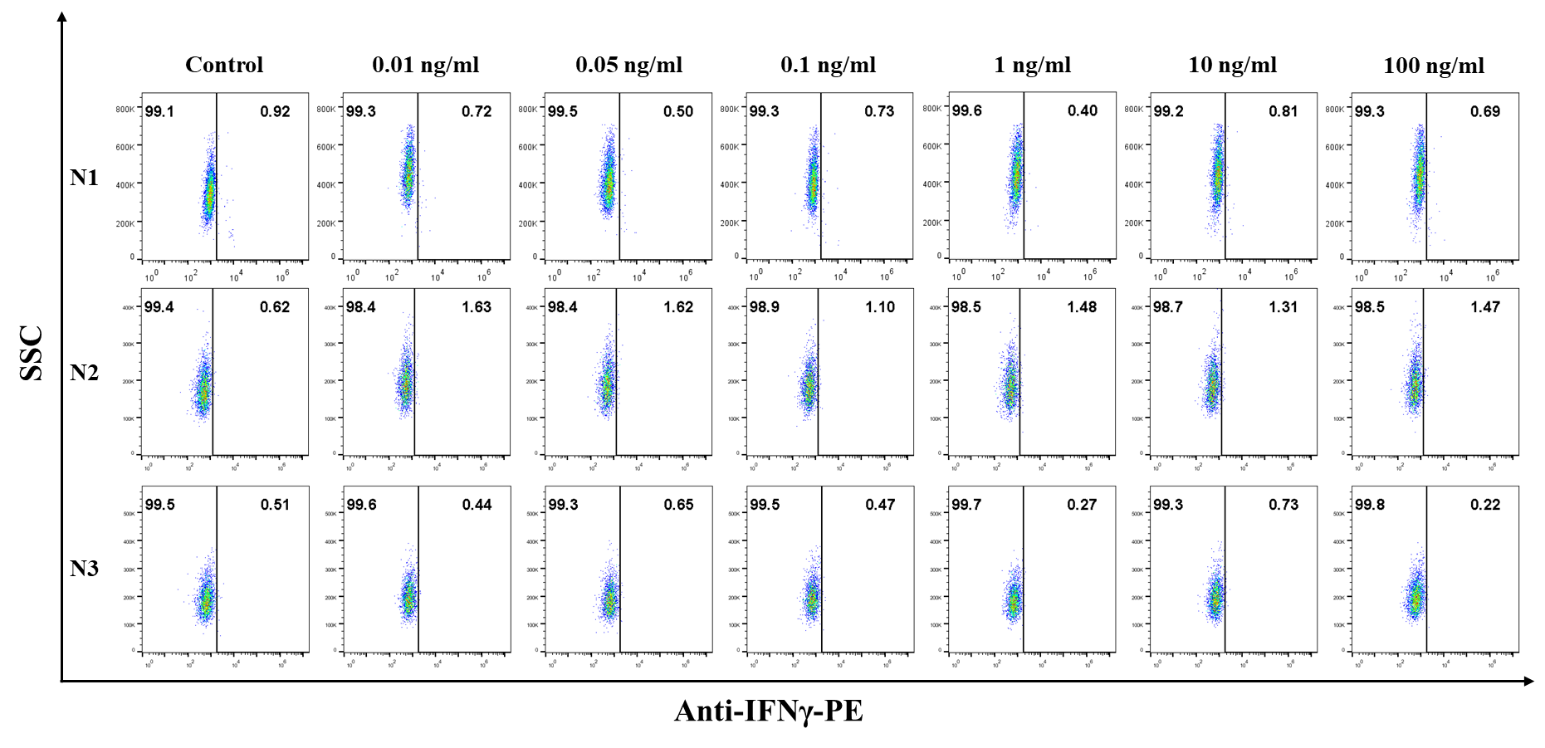


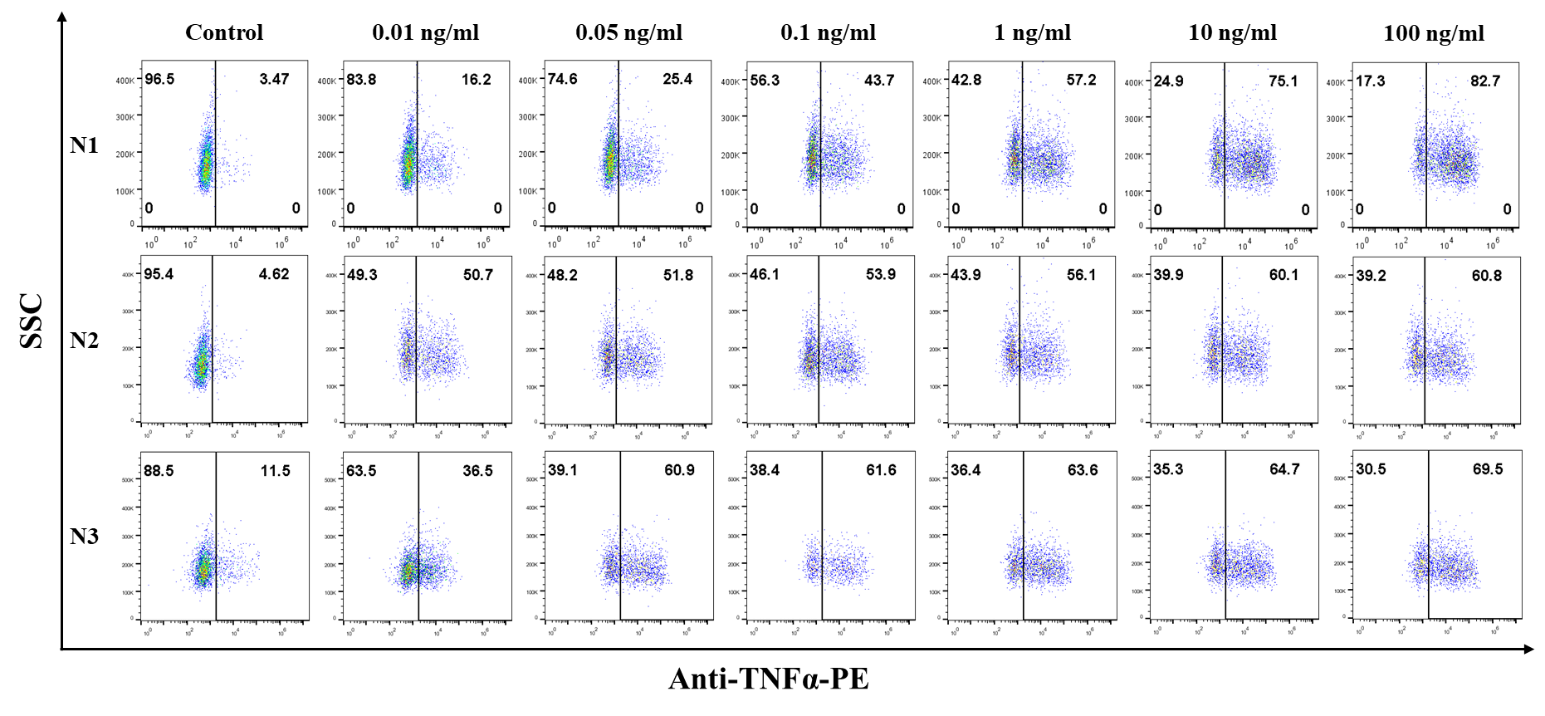


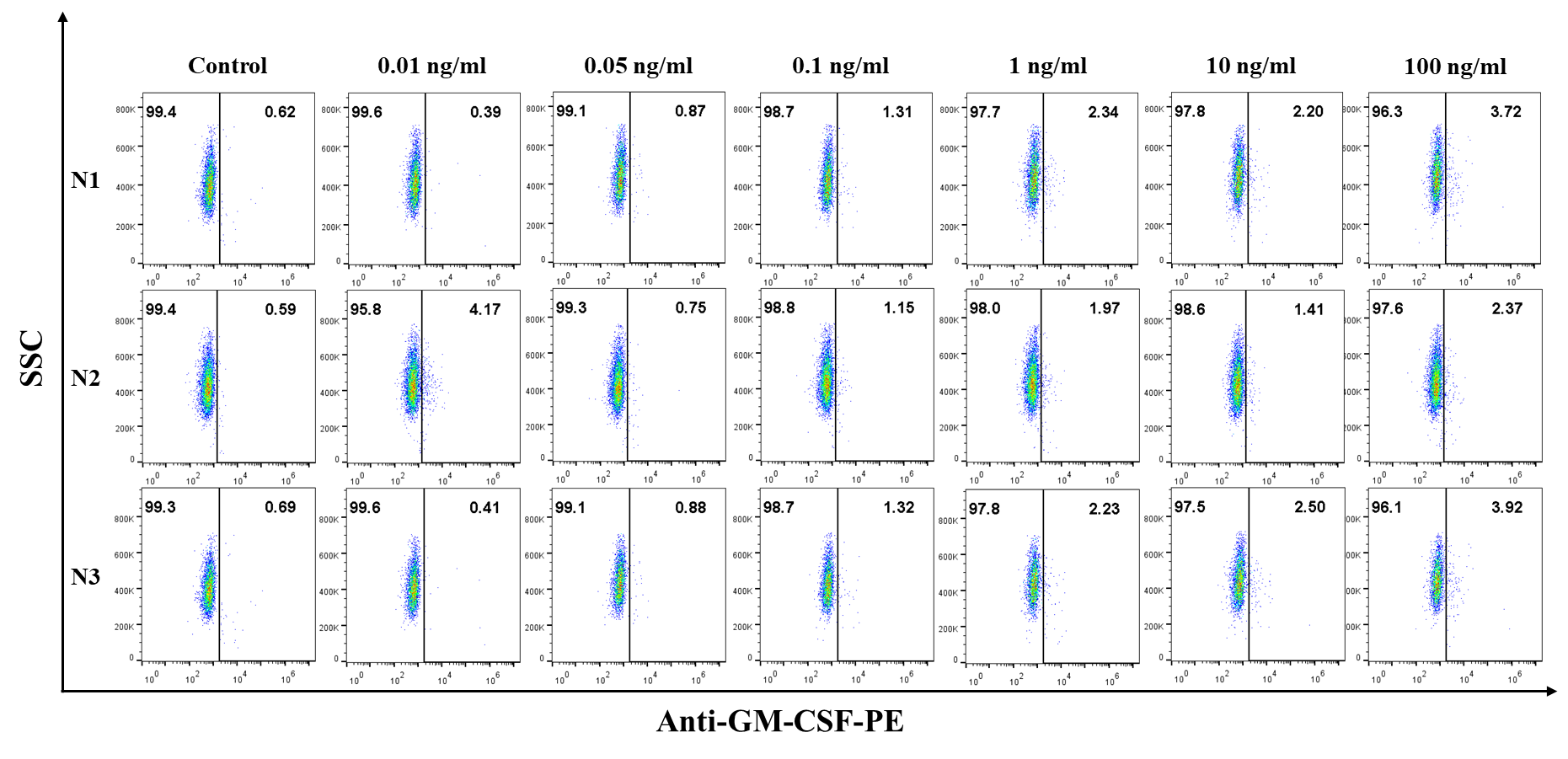


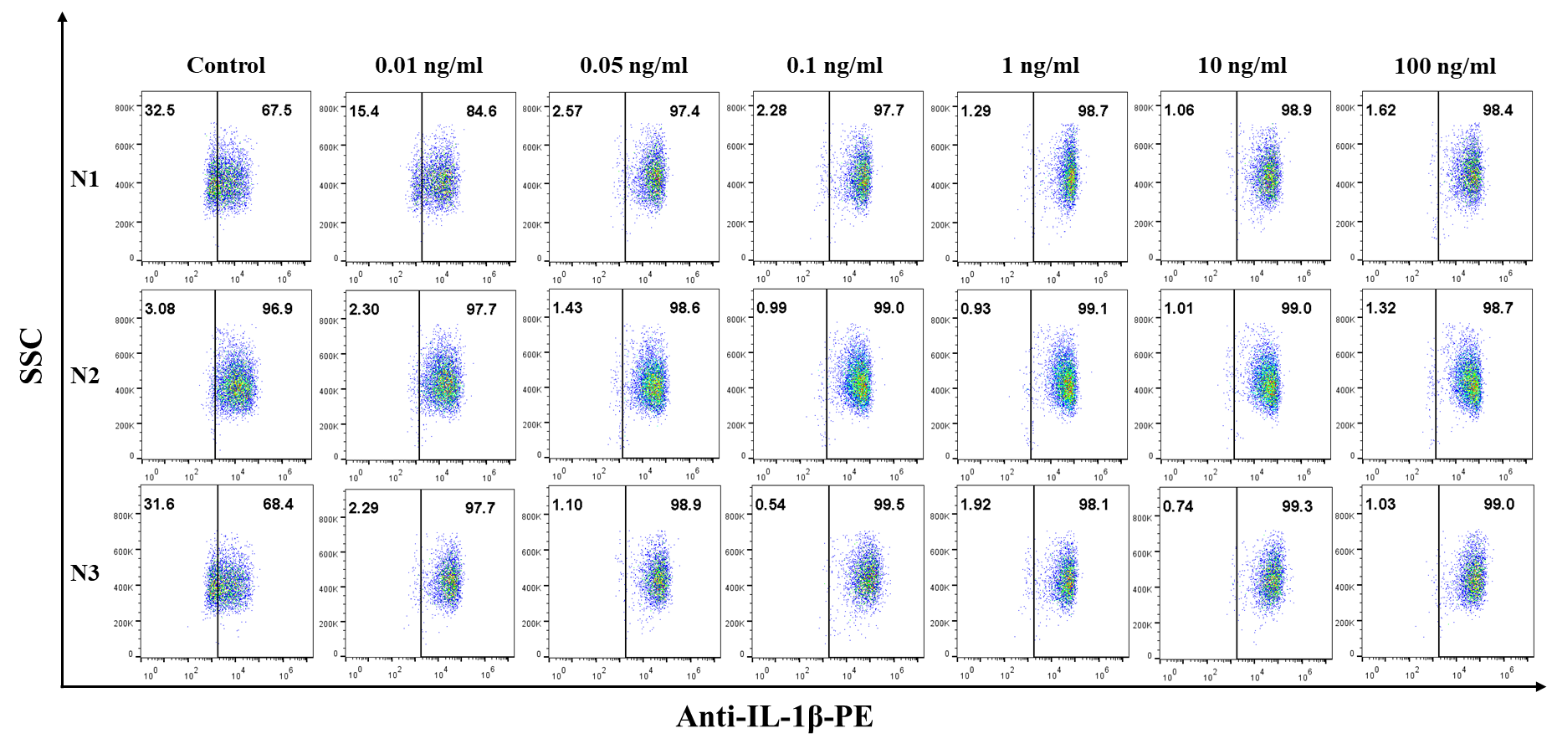


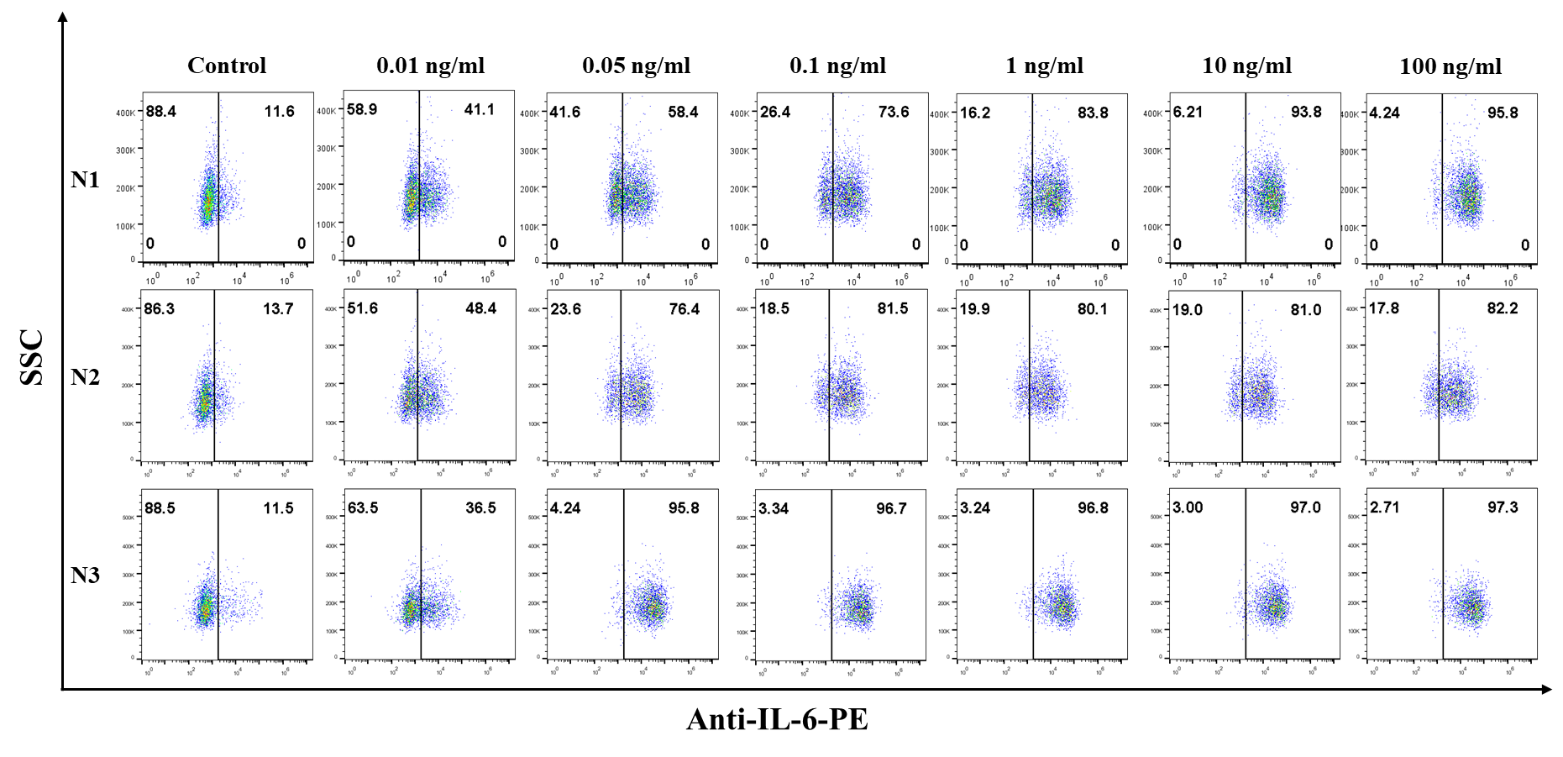


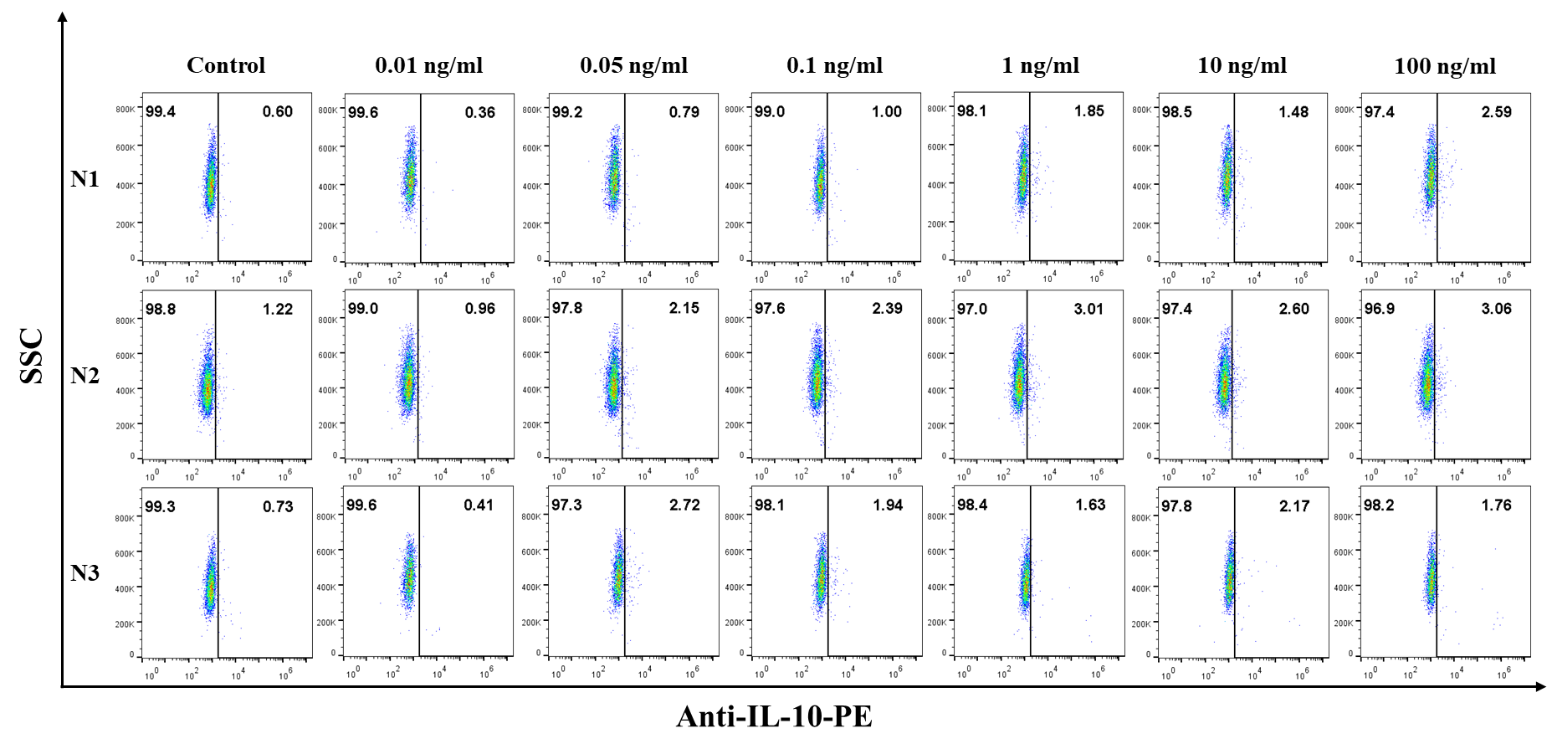


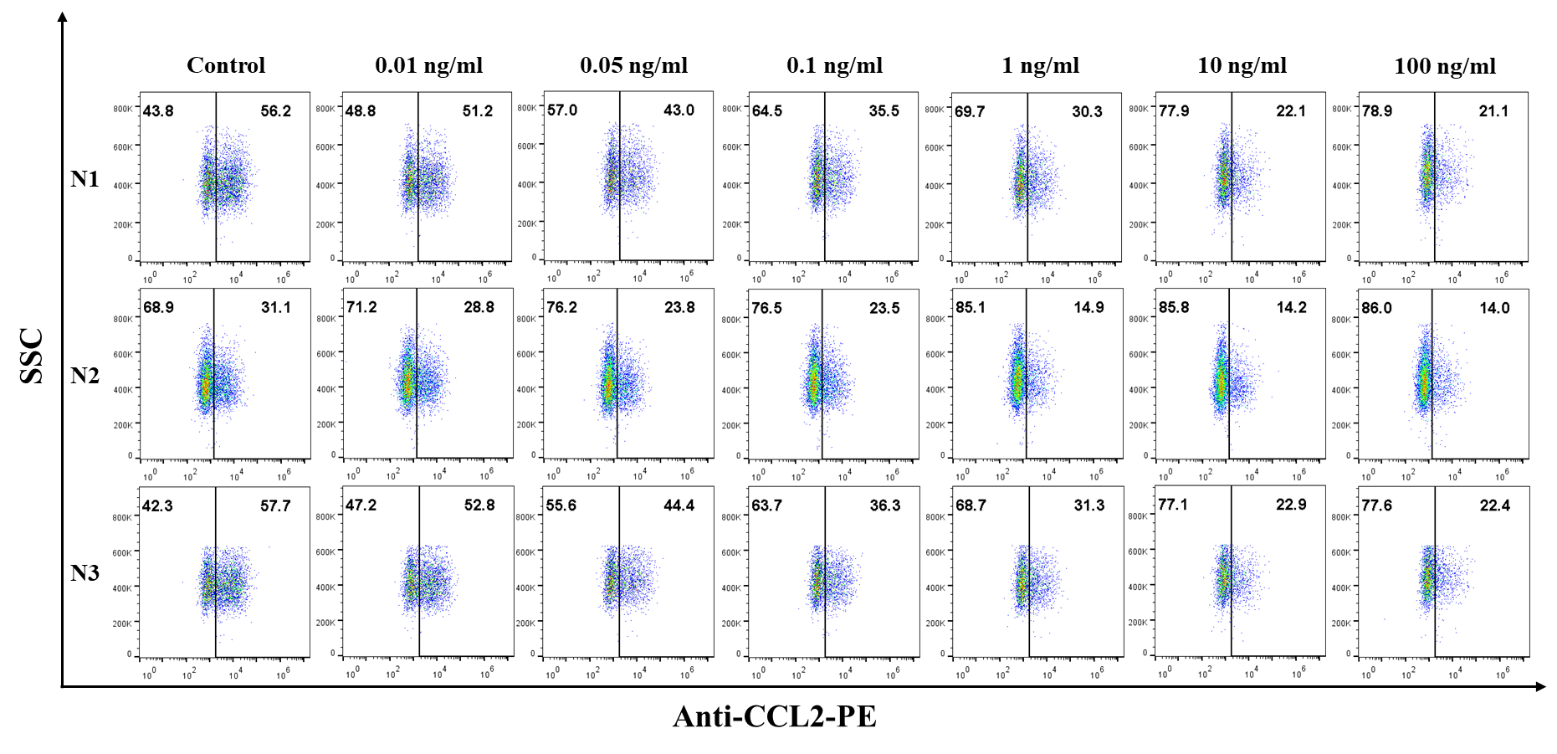


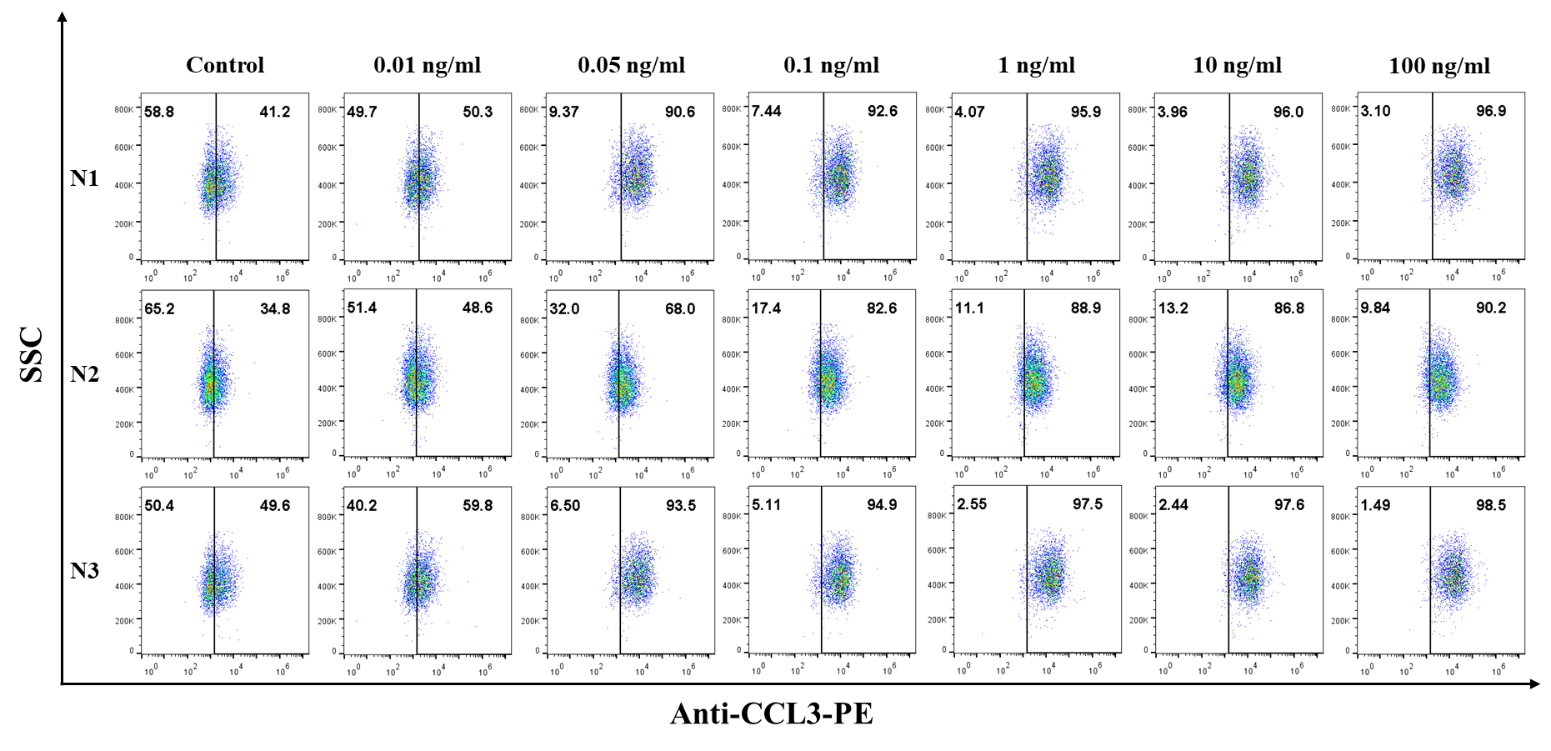


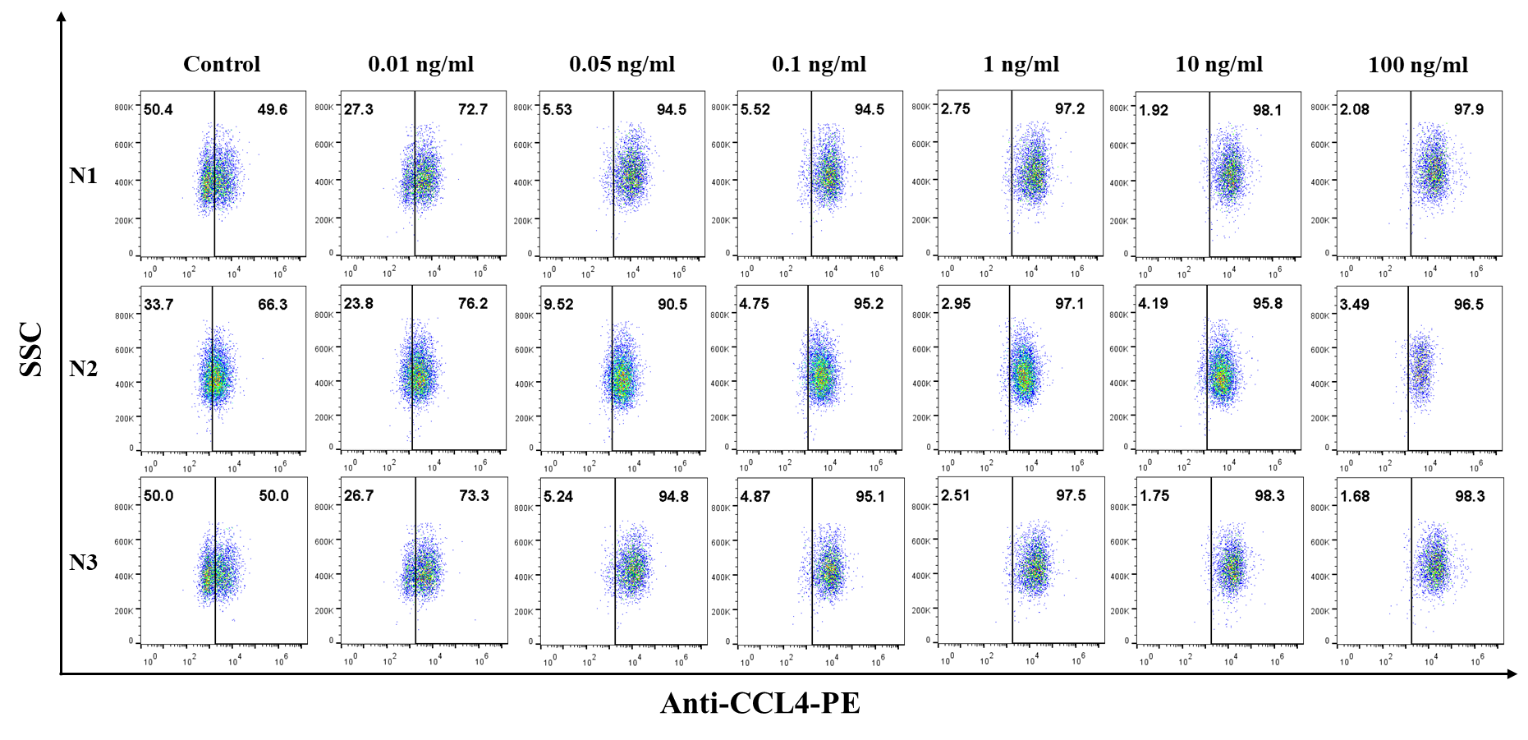


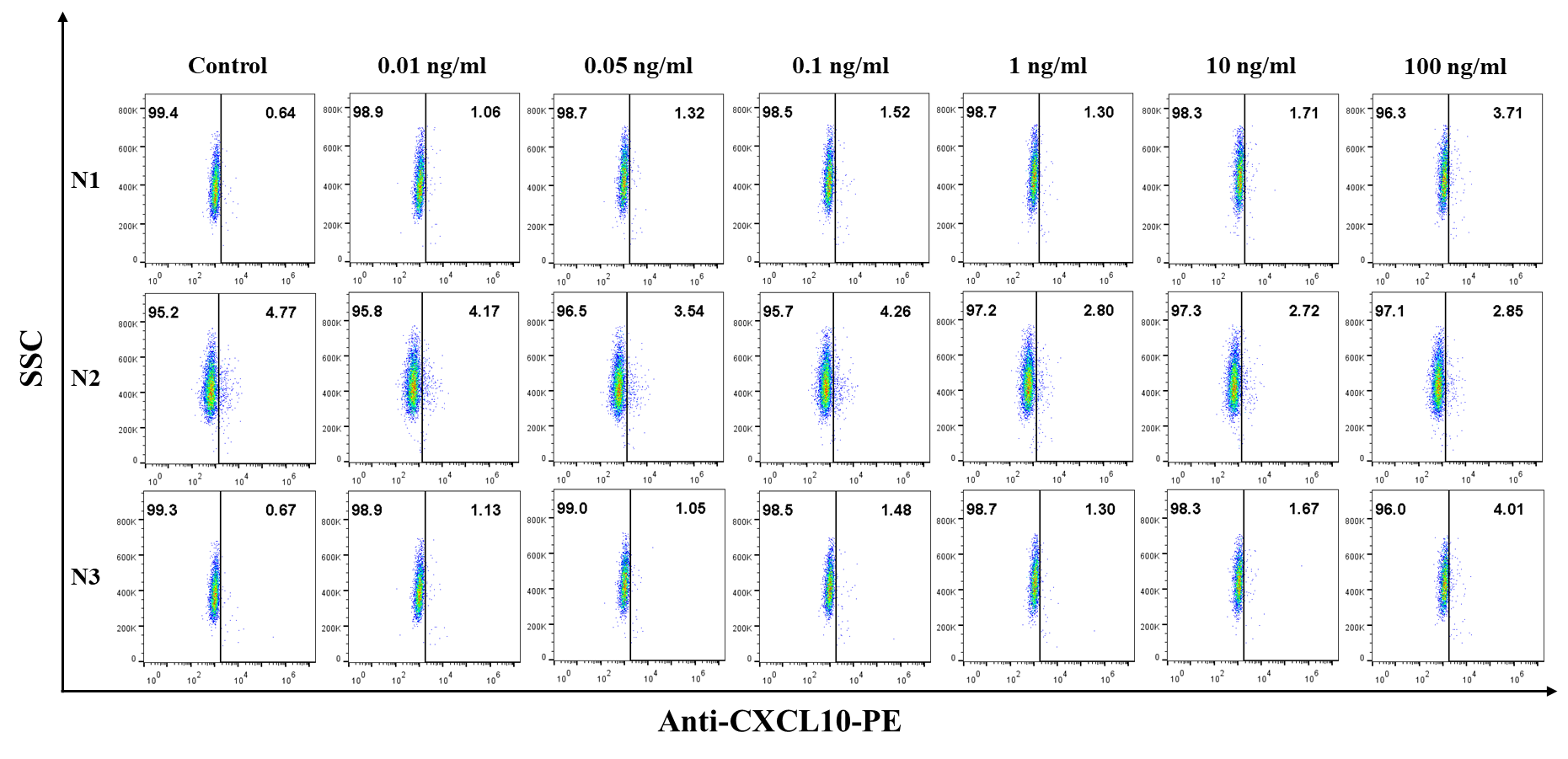


**Figure S1. Flow cytometric profiles of each subject (According to figure 1 in the paper):**

**Lipopolysaccharide induces the production of various cytokines and chemokines in monocytes.** PBMCs were stimulated with the indicated concentrations of LPS or in the absence of LPS (Control). The intracellular cytokines and chemokines were determined by flow cytometry. CD14+ monocyte population of the three individuals (as indicated) were gated and dot plotted on the expression of the indicated cytokines and chemokines are shown (numbers indicate the % cells).
