# Supplementary material for: Very low concentration of lipopolysaccharide can induce the production of various cytokines and chemokines in human primary monocytes
Source: BMC Res Notes. 2022 Feb 10;15:42. doi: 10.1186/s13104-022-05941-4 (PMC8832778; doi:10.1186/s13104-022-05941-4)
Supplement: Supplementary file 2 — Additional file 2: Figure S2. Flow cytometric profiles of each subject (according to Fig. 2 in the paper): the production of cytokines and chemokines in T lymphocytes upon lipopolysaccharide activation. PBMCs (n = 3) were stimulated with the indicated concentrations of LPS or in the absence of LPS (Control). Intracellular cytokines and chemokines were determined using flow cytometry. CD3+ T lymphocyte population of the three individuals (as indicated) were gated and dot plotted on the expression of the indicated cytokines and chemokines are shown (numbers indicate the % cells). [file 13104_2022_5941_MOESM2_ESM.docx]

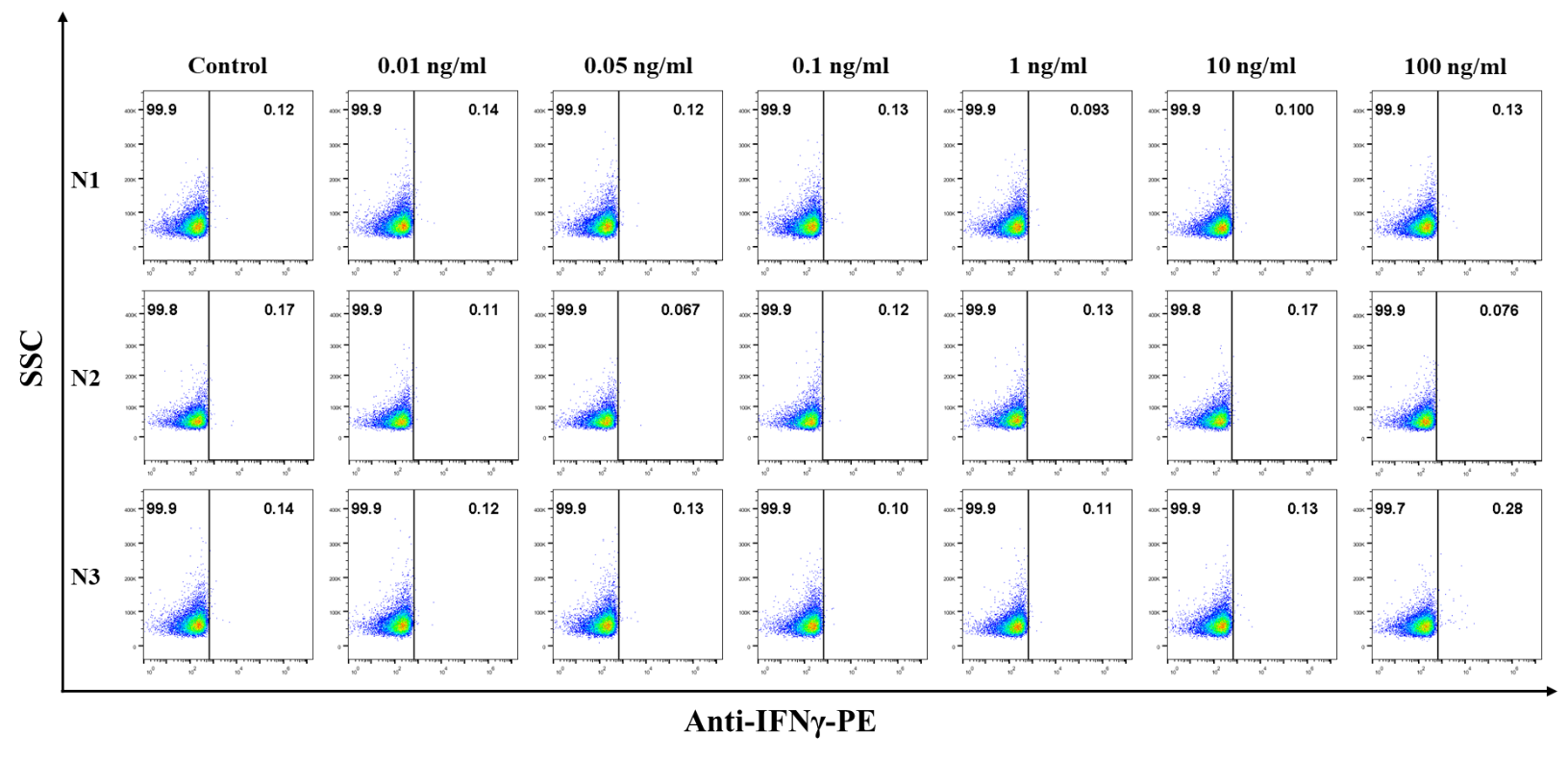


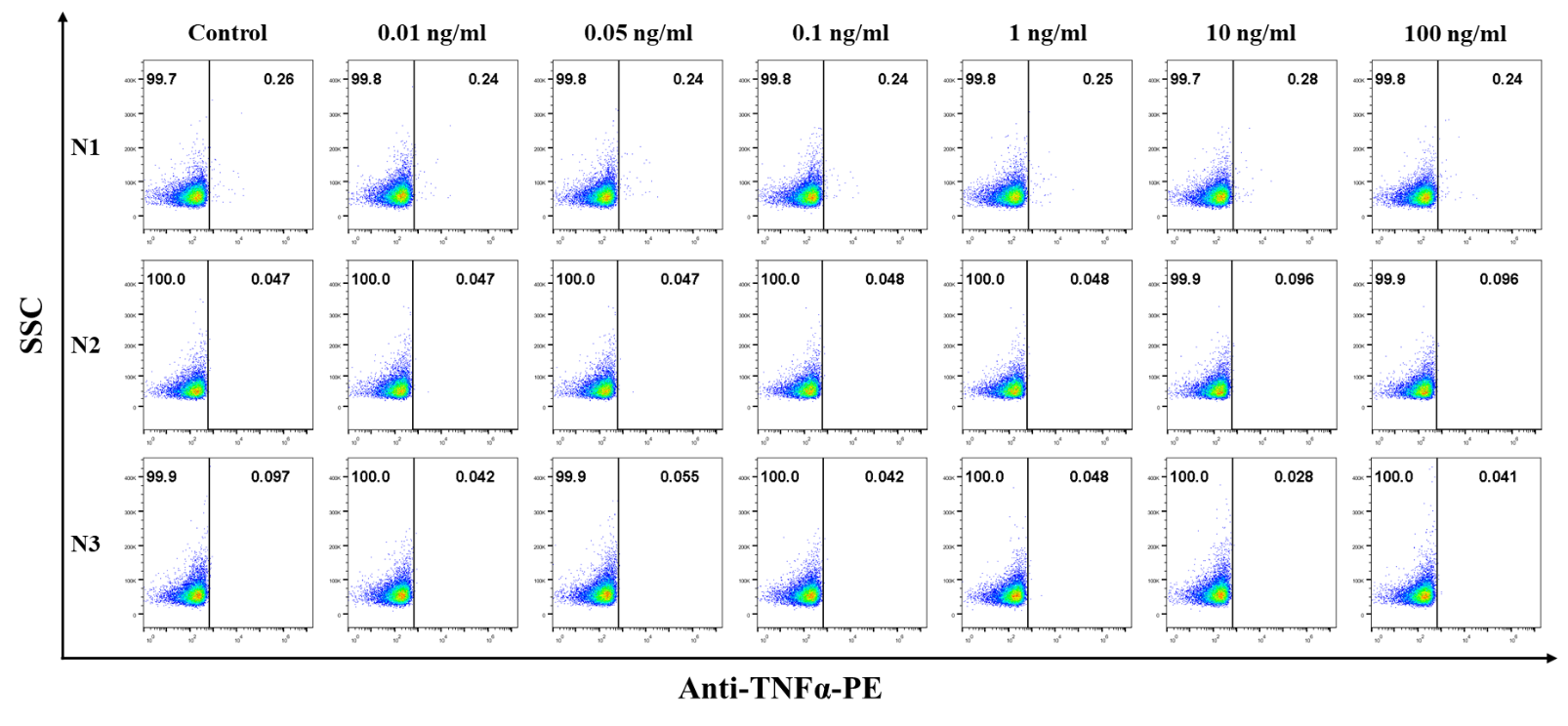


LPS induce cytokines and chemokines production in **T cells**


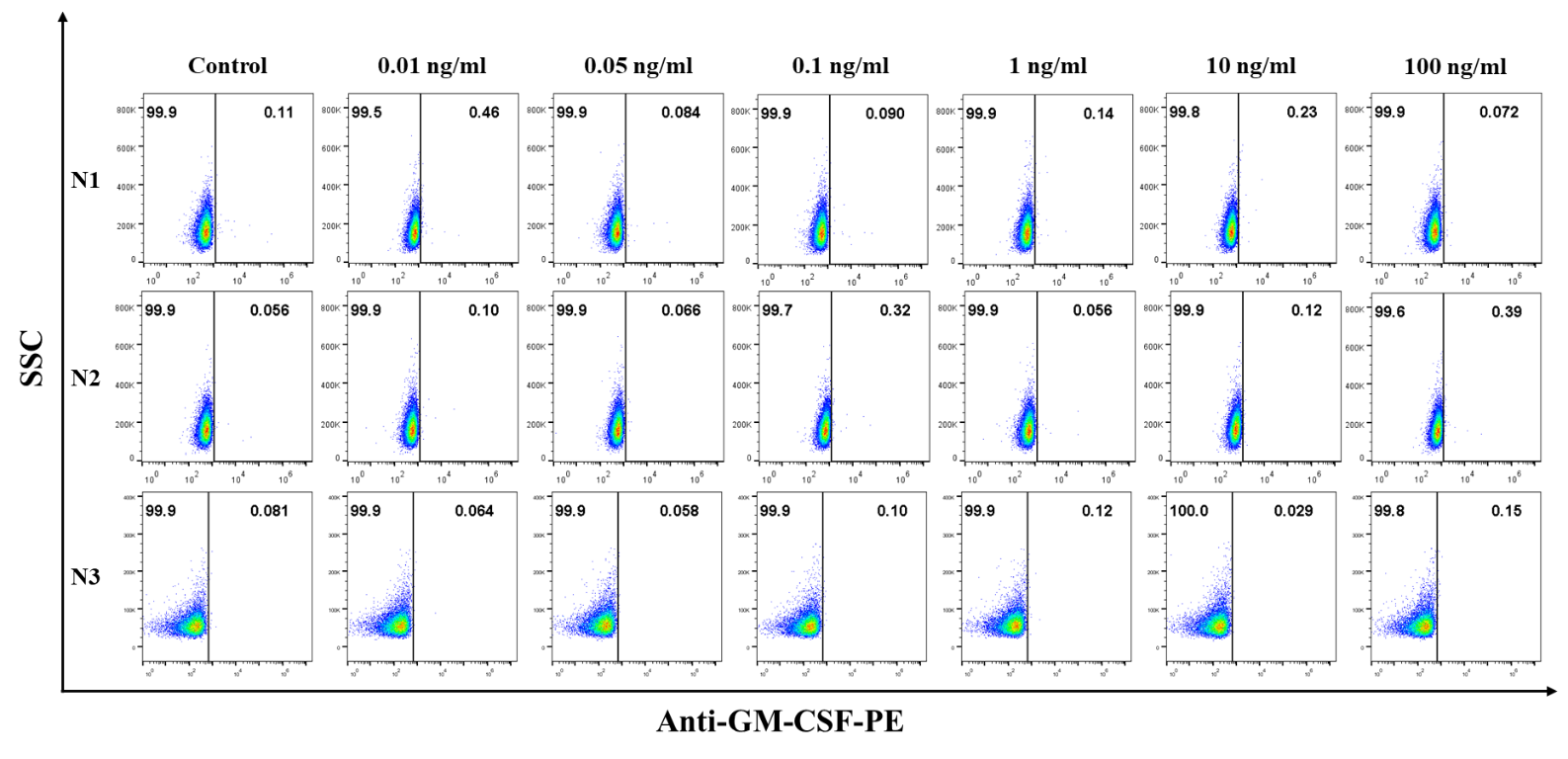


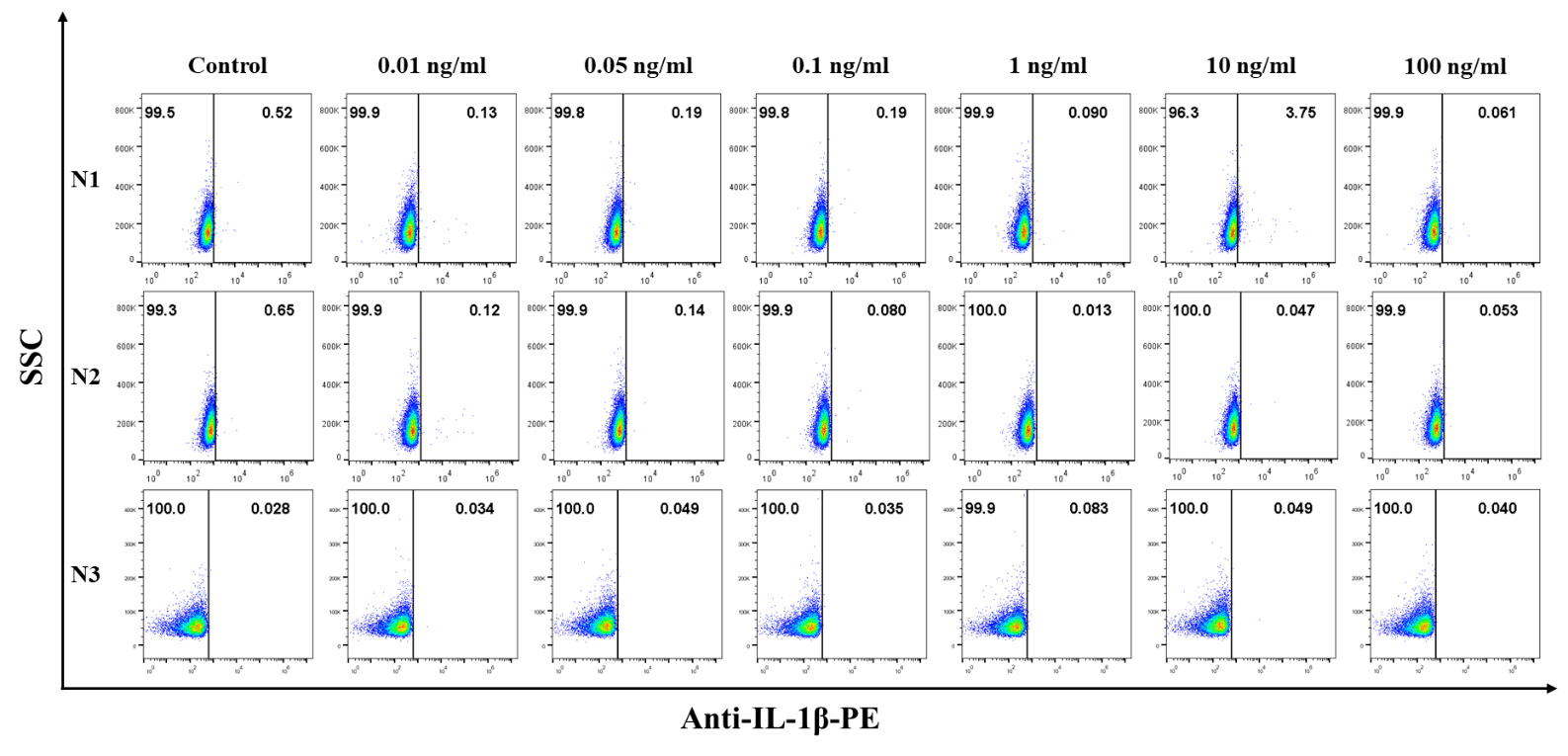


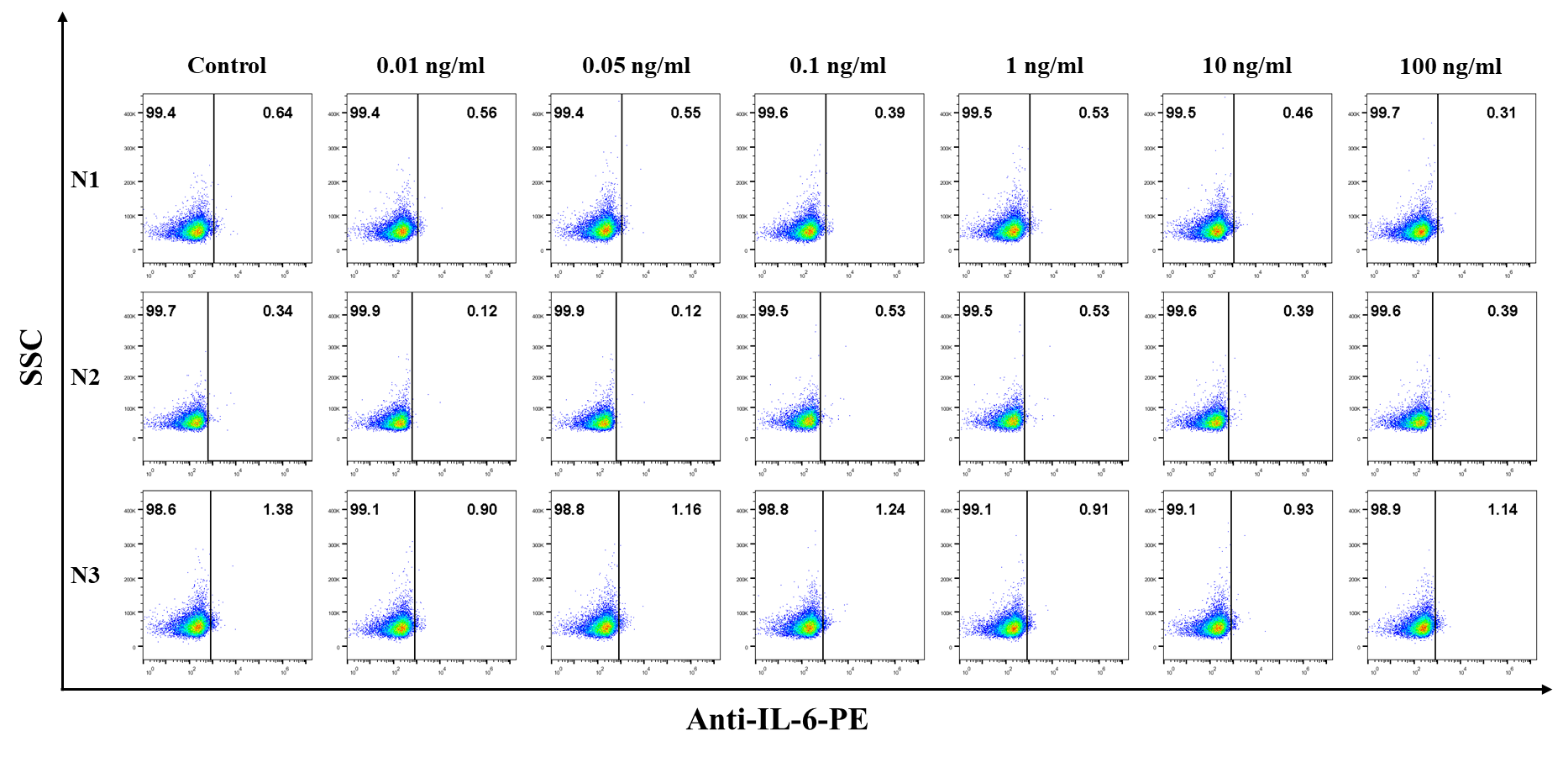


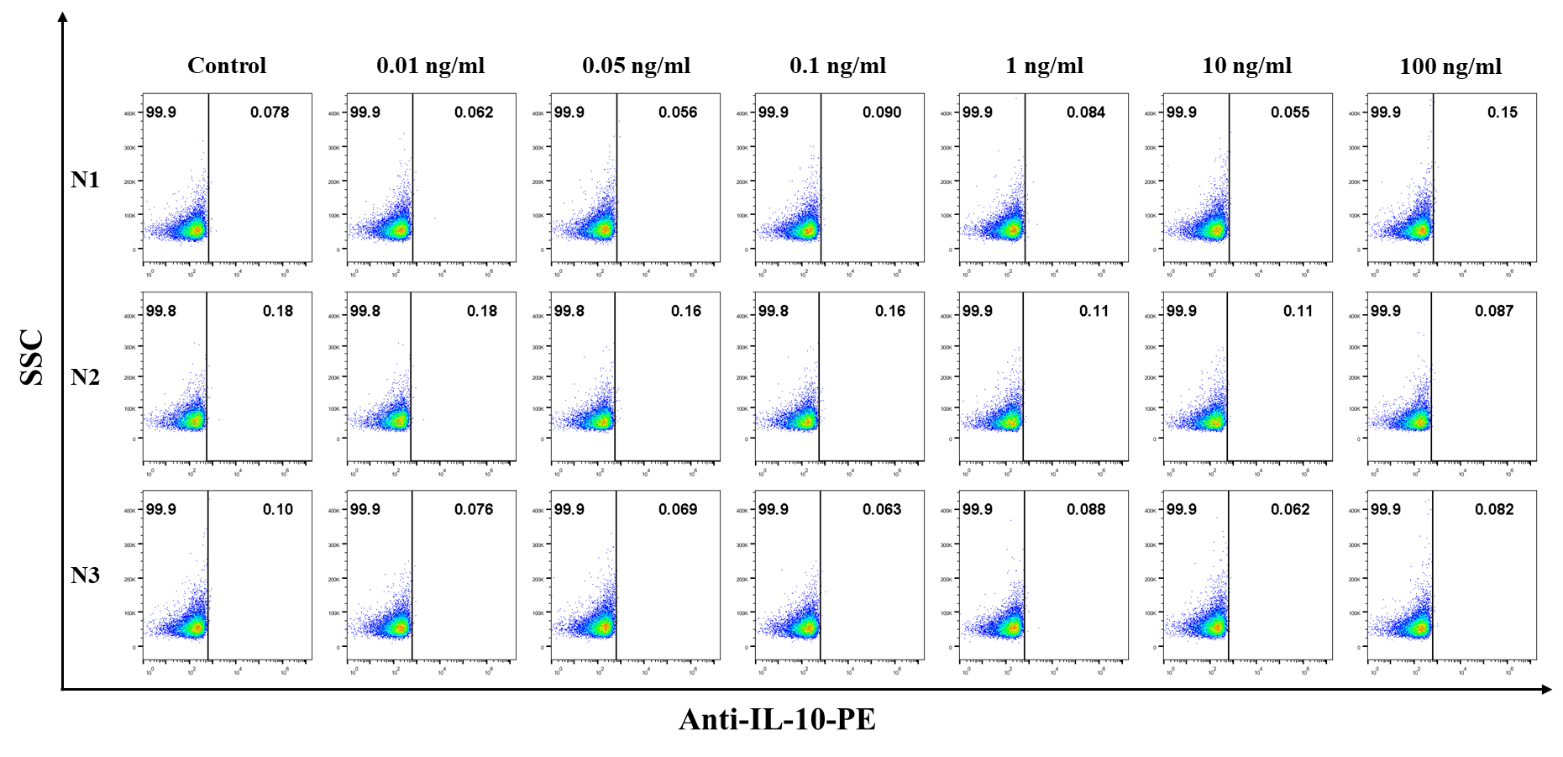


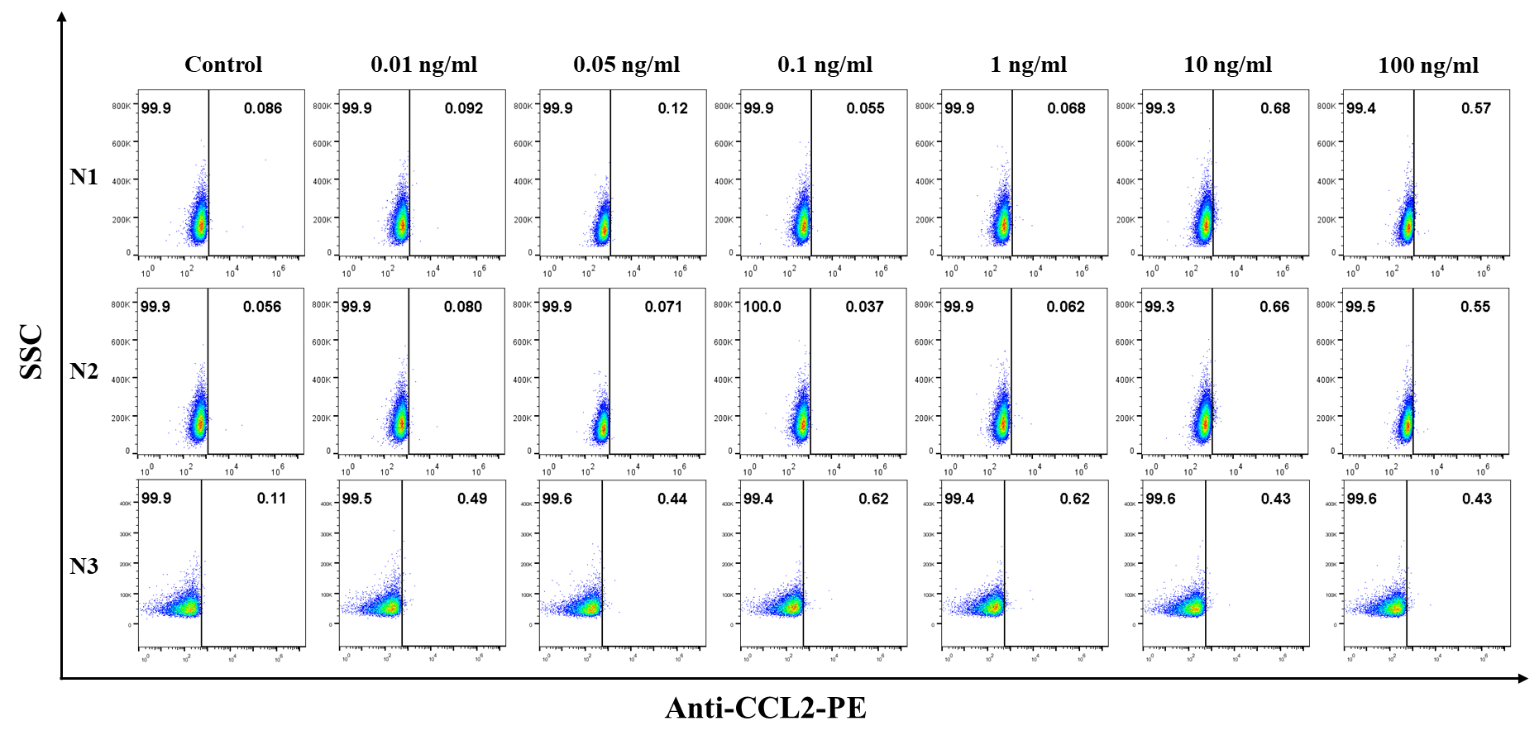


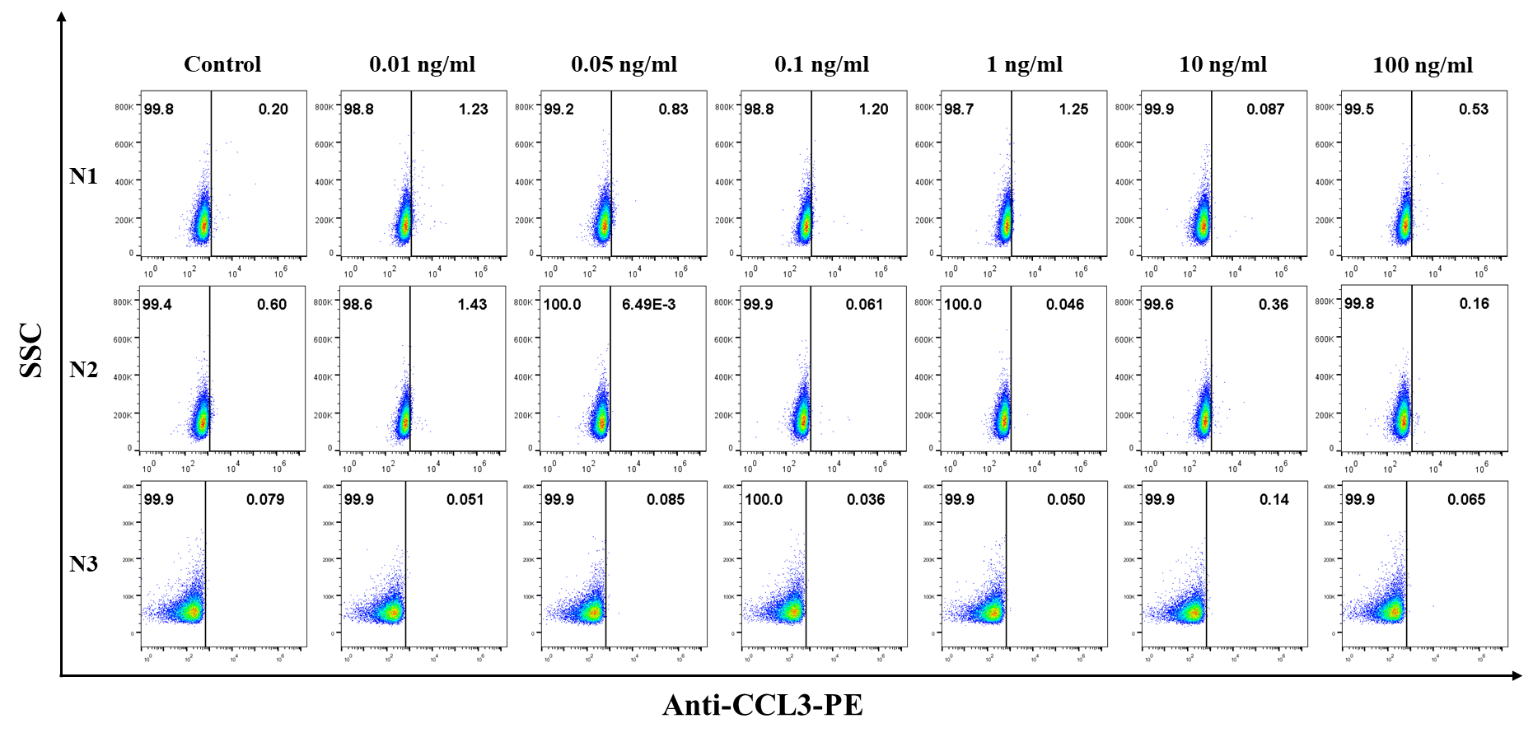


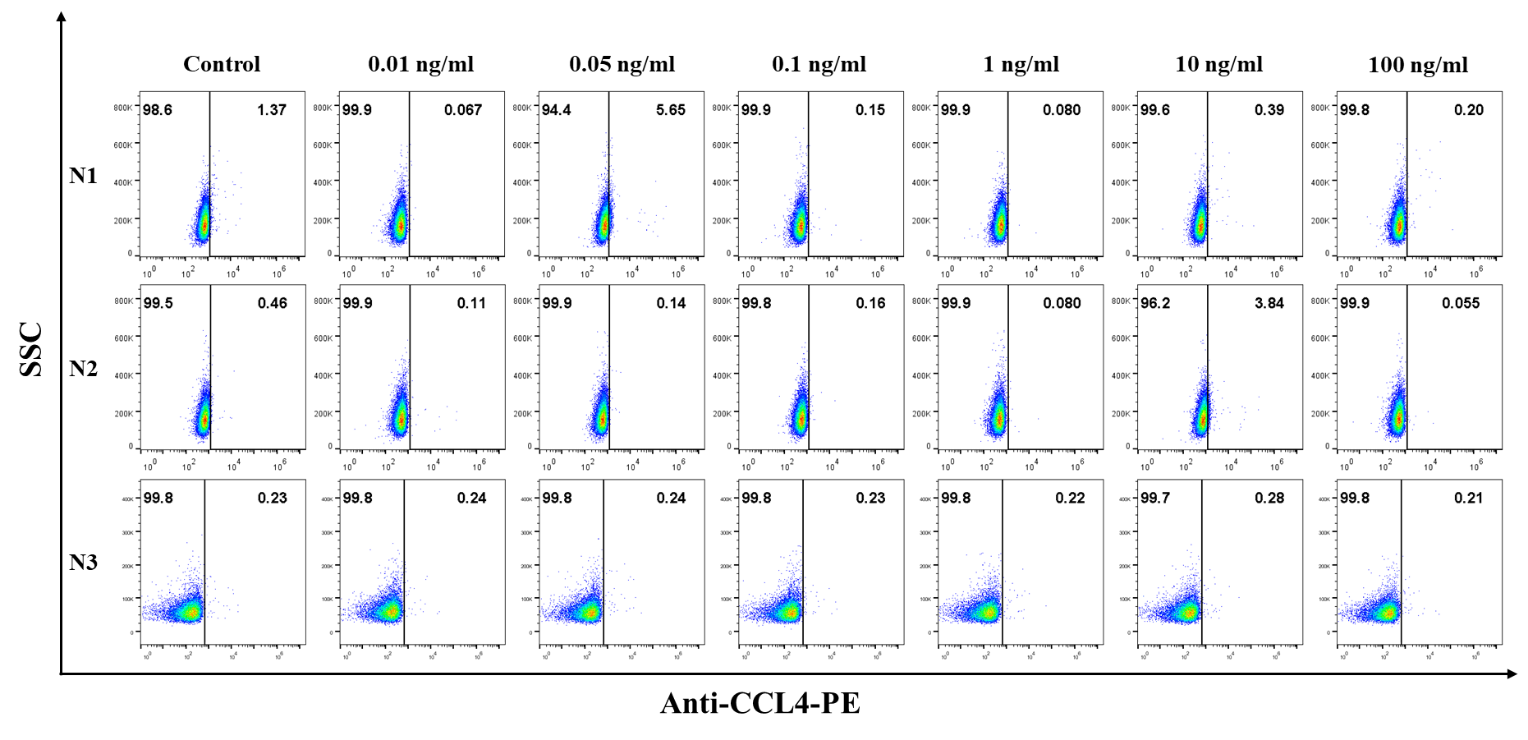


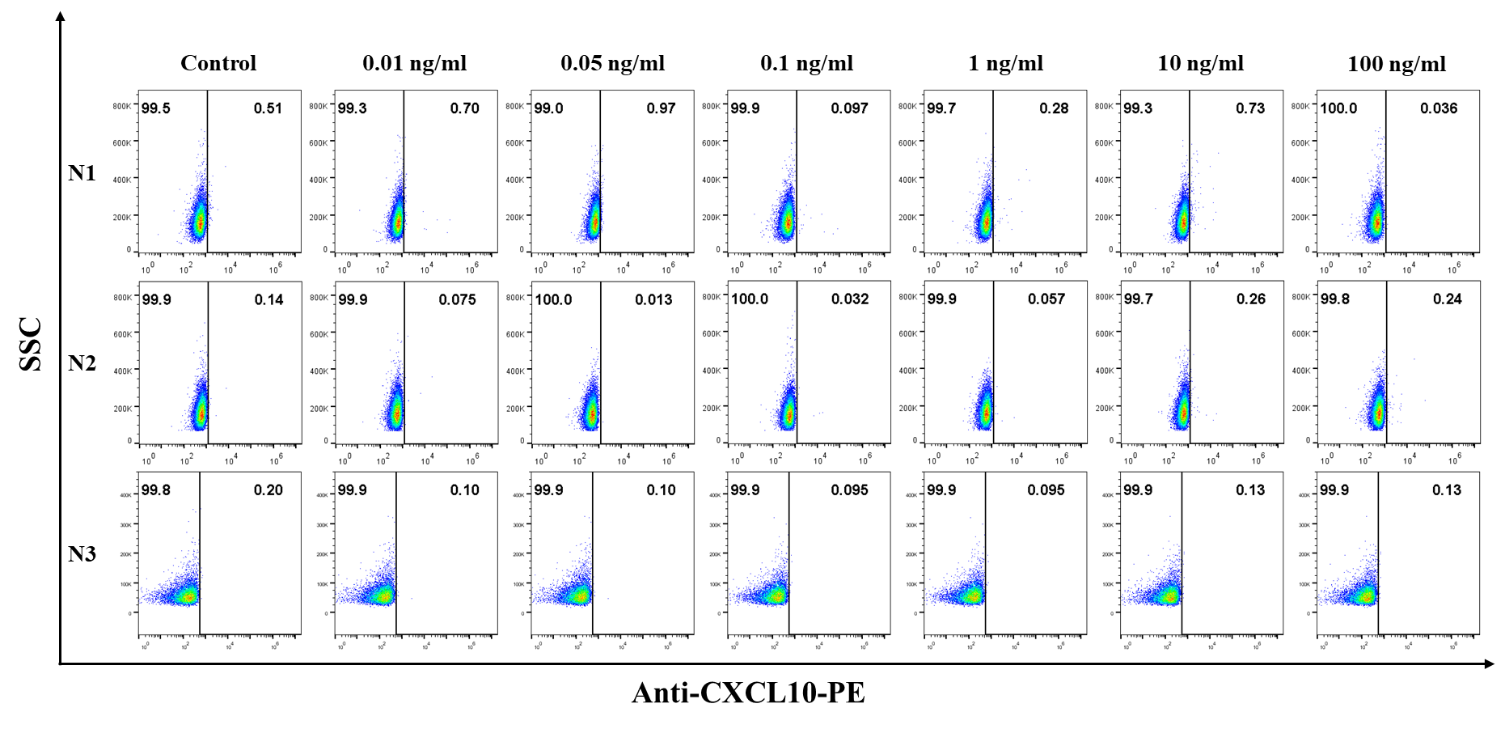


**Figure S2. Flow cytometric profiles of each subject (According to Figure 2 in the paper):**

**The production of cytokines and chemokines in T lymphocytes upon lipopolysaccharide activation.** PBMCs (n=3) were stimulated with the indicated concentrations of LPS or in the absence of LPS (Control). Intracellular cytokines and chemokines were determined using flow cytometry. CD3+ T lymphocyte population of the three individuals (as indicated) were gated and dot plotted on the expression of the indicated cytokines and chemokines are shown (numbers indicate the % cells).
