# Supplementary material for: Very low concentration of lipopolysaccharide can induce the production of various cytokines and chemokines in human primary monocytes
Source: BMC Res Notes. 2022 Feb 10;15:42. doi: 10.1186/s13104-022-05941-4 (PMC8832778; doi:10.1186/s13104-022-05941-4)
Supplement: Supplementary file 3 — Additional file 3: Figure S3. Flow cytometric profiles of each subject (according to Fig. 2 in the paper): the production of TNF-α and IFN-γ in T lymphocytes upon anti-CD3 and anti-CD28 mAbs activation. PBMCs were stimulated with anti-CD3 and anti-CD28 mAbs or or unstimulated control (as indicated). Intracellular cytokines were determined using flow cytometry. CD3+ T lymphocyte population of the three individuals (as indicated) were gated and dot plotted on the expression of the TNF-α and IFN-γ upon stimulation with anti-CD3 and anti-CD28 mAbs or unstimulated control are shown (numbers indicate the % cells). [file 13104_2022_5941_MOESM3_ESM.docx]

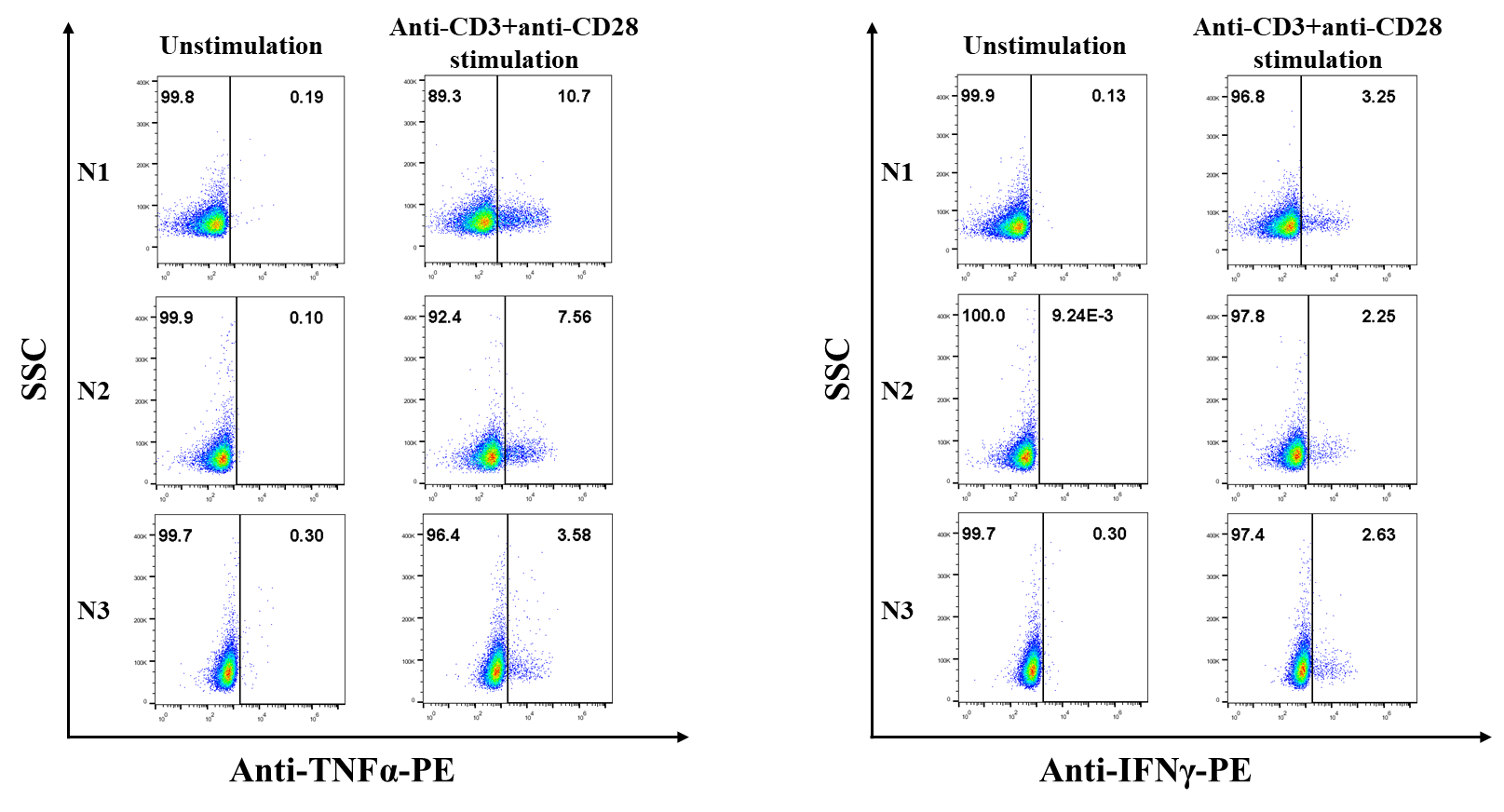


**Figure S3. Flow cytometric profiles of each subject (According to Figure 2 in the paper):**

**The production of TNF-α and IFN-γ in T lymphocytes upon anti-CD3 and anti-CD28 mAbs activation.** PBMCs were stimulated with anti-CD3 and anti-CD28 mAbs or or unstimulated control (as indicated). Intracellular cytokines were determined using flow cytometry. CD3+ T lymphocyte population of the three individuals (as indicated) were gated and dot plotted on the expression of the TNF-α and IFN-γ upon stimulation with anti-CD3 and anti-CD28 mAbs or unstimulated control are shown (numbers indicate the % cells).
