# Supplementary material for: Very low concentration of lipopolysaccharide can induce the production of various cytokines and chemokines in human primary monocytes
Source: BMC Res Notes. 2022 Feb 10;15:42. doi: 10.1186/s13104-022-05941-4 (PMC8832778; doi:10.1186/s13104-022-05941-4)
Supplement: Supplementary file 4 — Additional file 4: Figure S4. Flow cytometric profiles of each subject (according to Fig. 3 in the paper): lipopolysaccharide induces the production of TNF-α and IL-6 in monocytes. PBMCs were stimulated with the indicated concentrations of LPS or in the absence of LPS (Control). The intracellular TNF-α and IL-6 were determined by flow cytometry. CD14+ monocyte population of the three individuals (as indicated) were gated and dot plotted on the expression of the indicated cytokines are shown (numbers indicate the % cells). [file 13104_2022_5941_MOESM4_ESM.docx]

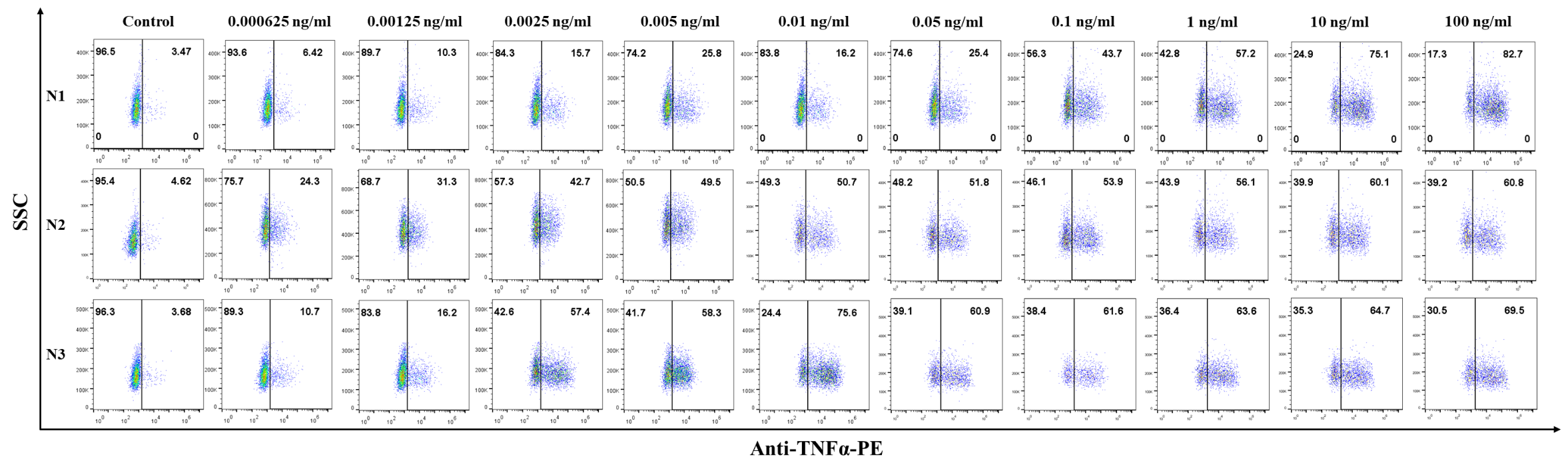


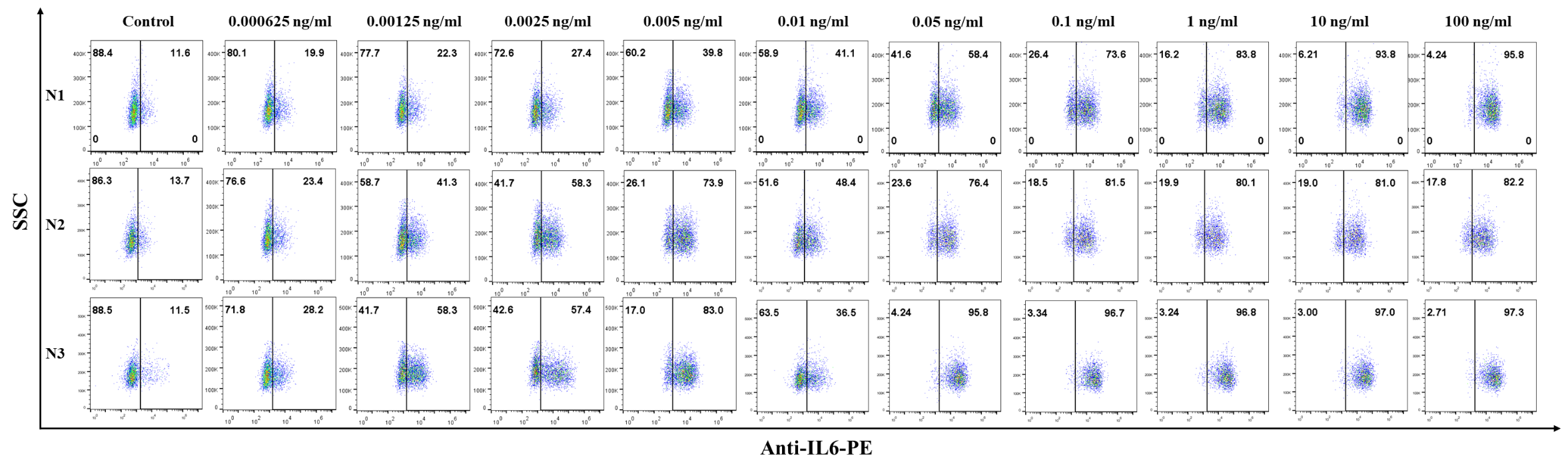


**Figure S4. Flow cytometric profiles of each subject (According to figure 3 in the paper):**

**Lipopolysaccharide induces the production of TNF-α and IL-6** **in monocytes.** PBMCs were stimulated with the indicated concentrations of LPS or in the absence of LPS (Control). The intracellular TNF-α and IL-6 were determined by flow cytometry. CD14+ monocyte population of the three individuals (as indicated) were gated and dot plotted on the expression of the indicated cytokines are shown (numbers indicate the % cells).
