# Supplementary material for: Very low concentration of lipopolysaccharide can induce the production of various cytokines and chemokines in human primary monocytes
Source: BMC Res Notes. 2022 Feb 10;15:42. doi: 10.1186/s13104-022-05941-4 (PMC8832778; doi:10.1186/s13104-022-05941-4)
Supplement: Supplementary file 5 — Additional file 5: Table S1. Data analyzed form flow cytometric profiles of each subject (according to Fig. 1 in the paper): lipopolysaccharide induces the production of various cytokines and chemokines in monocytes. PBMCs were stimulated with the indicated concentrations of LPS. The intracellular cytokines and chemokines were determined by flow cytometry. CD14+ monocyte population of the three individuals (as indicated) were gated and mean fluorescence intensity of the expression of the indicated cytokines and chemokines are shown. [file 13104_2022_5941_MOESM5_ESM.docx]

**Table S1. Data analyzed form flow cytometric profiles of each subject (According to figure 1 in the paper):**

**Lipopolysaccharide induces the production of various cytokines and chemokines in monocytes.** PBMCs were stimulated with the indicated concentrations of LPS. The intracellular cytokines and chemokines were determined by flow cytometry. CD14+ monocyte population of the three individuals (as indicated) were gated and mean fluorescence intensity of the expression of the indicated cytokines and chemokines are shown.

| Mean Fluorescence Intensity (MFI) | | | |
| --- | --- | --- | --- |
| IFN-gamma | | | |
| LPS (ng/ml) | N1 | N2 | N3 |
| 0 | 487 | 902 | 695 |
| 0.01 | 509 | 842 | 675 |
| 0.05 | 566 | 802 | 684 |
| 0.1 | 547 | 857 | 700 |
| 1 | 508 | 827 | 677 |
| 10 | 520 | 837 | 678 |
| 100 | 535 | 874 | 698 |
|  |  |  |  |
|  |  |  |  |
| Mean Fluorescence Intensity (MFI) | | | |
| TNF-alpha | | | |
| LPS (ng/ml) | N1 | N2 | N3 |
| 0 | 481 | 706 | 600 |
| 0.01 | 884 | 2510 | 1698 |
| 0.05 | 2052 | 3288 | 2680 |
| 0.1 | 2258 | 4523 | 3400 |
| 1 | 2661 | 5080 | 3875 |
| 10 | 3108 | 5129 | 4125 |
| 100 | 3162 | 5891 | 4589 |
|  |  |  |  |
|  |  |  |  |
|  |  |  |  |
| Mean Fluorescence Intensity (MFI) | | | |
| GM-CSF | | | |
| LPS (ng/ml) | N1 | N2 | N3 |
| 0 | 667 | 534 | 603 |
| 0.01 | 680 | 504 | 599 |
| 0.05 | 690 | 536 | 618 |
| 0.1 | 691 | 563 | 629 |
| 1 | 687 | 580 | 634 |
| 10 | 697 | 594 | 650 |
| 100 | 690 | 625 | 665 |
|  |  |  |  |
|  |  |  |  |
| Mean Fluorescence Intensity (MFI) | | | |
| IL1-beta | | | |
| LPS (ng/ml) | N1 | N2 | N3 |
| 0 | 10734 | 9806 | 10200 |
| 0.01 | 21895 | 13433 | 17890 |
| 0.05 | 30569 | 22220 | 26398 |
| 0.1 | 28242 | 27902 | 28120 |
| 1 | 30879 | 32997 | 32015 |
| 10 | 30843 | 32066 | 32547 |
| 100 | 36162 | 33768 | 35065 |
|  |  |  |  |
|  |  |  |  |
| Mean Fluorescence Intensity (MFI) | | | |
| IL-6 | | | |
| LPS (ng/ml) | N1 | N2 | N3 |
| 0 | 610 | 756 | 683 |
| 0.01 | 1378 | 1786 | 1682 |
| 0.05 | 3405 | 3655 | 3634 |
| 0.1 | 3784 | 4023 | 3945 |
| 1 | 4060 | 4597 | 4258 |
| 10 | 4350 | 4729 | 4655 |
| 100 | 4582 | 6257 | 5528 |
|  |  |  |  |
|  |  |  |  |
| Mean Fluorescence Intensity (MFI) | | | |
| IL-10 | | | |
| LPS (ng/ml) | N1 | N2 | N3 |
| 0 | 844 | 609 | 729 |
| 0.01 | 895 | 524 | 711 |
| 0.05 | 825 | 591 | 719 |
| 0.1 | 884 | 622 | 745 |
| 1 | 920 | 631 | 772 |
| 10 | 859 | 647 | 753 |
| 100 | 942 | 680 | 780 |

| Mean Fluorescence Intensity (MFI) | | | |
| --- | --- | --- | --- |
| CCL2 | | | |
| LPS (ng/ml) | N1 | N2 | N3 |
| 0 | 1434 | 1064 | 1252 |
| 0.01 | 1228 | 968 | 1102 |
| 0.05 | 1058 | 904 | 999 |
| 0.1 | 1034 | 894 | 987 |
| 1 | 966 | 748 | 865 |
| 10 | 977 | 778 | 883 |
| 100 | 929 | 791 | 869 |
|  |  |  |  |
|  |  |  |  |
| Mean Fluorescence Intensity (MFI) | | | |
| CCL3 | | | |
| LPS (ng/ml) | N1 | N2 | N3 |
| 0 | 990 | 1120 | 1060 |
| 0.01 | 2689 | 1380 | 2036 |
| 0.05 | 4040 | 1870 | 2612 |
| 0.1 | 4720 | 2733 | 3572 |
| 1 | 4796 | 3317 | 3988 |
| 10 | 4539 | 3176 | 4215 |
| 100 | 4655 | 3730 | 4356 |
|  |  |  |  |
|  |  |  |  |
| Mean Fluorescence Intensity (MFI) | | | |
| CCL4 | | | |
| LPS (ng/ml) | N1 | N2 | N3 |
| 0 | 1367 | 1857 | 1645 |
| 0.01 | 3961 | 2418 | 3389 |
| 0.05 | 6396 | 3428 | 4956 |
| 0.1 | 7120 | 4854 | 5867 |
| 1 | 7245 | 5611 | 6385 |
| 10 | 7522 | 5464 | 6721 |
| 100 | 8652 | 5603 | 7217 |
|  |  |  |  |
|  |  |  |  |
| Mean Fluorescence Intensity (MFI) | | | |
| CXCL10 | | | |
| LPS (ng/ml) | N1 | N2 | N3 |
| 0 | 679 | 647 | 673 |
| 0.01 | 691 | 620 | 665 |
| 0.05 | 707 | 618 | 662 |
| 0.1 | 670 | 656 | 670 |
| 1 | 704 | 620 | 668 |
| 10 | 714 | 652 | 683 |
| 100 | 726 | 658 | 692 |
