# Supplementary material for: Very low concentration of lipopolysaccharide can induce the production of various cytokines and chemokines in human primary monocytes
Source: BMC Res Notes. 2022 Feb 10;15:42. doi: 10.1186/s13104-022-05941-4 (PMC8832778; doi:10.1186/s13104-022-05941-4)
Supplement: Supplementary file 6 — Additional file 6: Table S2. Data analyzed form flow cytometric profiles of each subject (according to Fig. 2 in the paper): lipopolysaccharide induces the production of various cytokines and chemokines in T lymphocytes. PBMCs were stimulated with the indicated concentrations of LPS. The intracellular cytokines and chemokines were determined by flow cytometry. CD3+ T lymphocyte population of the three individuals (as indicated) were gated and mean fluorescence intensity of the expression of the indicated cytokines and chemokines are shown. [file 13104_2022_5941_MOESM6_ESM.docx]

**Table S2. Data analyzed form flow cytometric profiles of each subject (According to figure 2 in the paper):**

**Lipopolysaccharide induces the production of various cytokines and chemokines in T lymphocytes.** PBMCs were stimulated with the indicated concentrations of LPS. The intracellular cytokines and chemokines were determined by flow cytometry. CD3+ T lymphocyte population of the three individuals (as indicated) were gated and mean fluorescence intensity of the expression of the indicated cytokines and chemokines are shown.

| Mean Fluorescence Intensity (MFI) | | | |
| --- | --- | --- | --- |
| IFN-gamma | | | |
| LPS (ng/ml) | N1 | N2 | N3 |
| 0 | 523 | 805 | 661 |
| 0.01 | 509 | 842 | 673 |
| 0.05 | 566 | 785 | 673 |
| 0.1 | 547 | 798 | 670 |
| 1 | 508 | 827 | 665 |
| 10 | 520 | 837 | 676 |
| 100 | 535 | 780 | 655 |
|  |  |  |  |
|  |  |  |  |
| Mean Fluorescence Intensity (MFI) | | | |
| TNF-alpha | | | |
| LPS (ng/ml) | N1 | N2 | N3 |
| 0 | 481 | 688 | 596 |
| 0.01 | 489 | 650 | 581 |
| 0.05 | 520 | 678 | 610 |
| 0.1 | 498 | 632 | 564 |
| 1 | 495 | 649 | 583 |
| 10 | 501 | 689 | 606 |
| 100 | 576 | 622 | 610 |
|  |  |  |  |
|  |  |  |  |
| Mean Fluorescence Intensity (MFI) | | | |
| GM-CSF | | | |
| LPS (ng/ml) | N1 | N2 | N3 |
| 0 | 667 | 534 | 604 |
| 0.01 | 680 | 504 | 595 |
| 0.05 | 659 | 536 | 601 |
| 0.1 | 691 | 563 | 630 |
| 1 | 623 | 580 | 605 |
| 10 | 697 | 594 | 649 |
| 100 | 666 | 625 | 629 |
|  |  |  |  |
|  |  |  |  |
| Mean Fluorescence Intensity (MFI) | | | |
| IL1-beta | | | |
| LPS (ng/ml) | N1 | N2 | N3 |
| 0 | 654 | 687 | 665 |
| 0.01 | 632 | 695 | 658 |
| 0.05 | 653 | 689 | 665 |
| 0.1 | 689 | 700 | 689 |
| 1 | 644 | 620 | 630 |
| 10 | 625 | 685 | 655 |
| 100 | 670 | 623 | 641 |
|  |  |  |  |
|  |  |  |  |
| Mean Fluorescence Intensity (MFI) | | | |
| IL-6 | | | |
| LPS (ng/ml) | N1 | N2 | N3 |
| 0 | 756 | 685 | 656 |
| 0.01 | 702 | 638 | 666 |
| 0.05 | 698 | 694 | 670 |
| 0.1 | 711 | 678 | 682 |
| 1 | 723 | 695 | 645 |
| 10 | 756 | 698 | 650 |
| 100 | 755 | 700 | 658 |
|  |  |  |  |
|  |  |  |  |
| Mean Fluorescence Intensity (MFI) | | | |
| IL-10 | | | |
| LPS (ng/ml) | N1 | N2 | N3 |
| 0 | 844 | 609 | 720 |
| 0.01 | 895 | 568 | 711 |
| 0.05 | 825 | 628 | 725 |
| 0.1 | 884 | 622 | 735 |
| 1 | 850 | 631 | 714 |
| 10 | 859 | 647 | 739 |
| 100 | 845 | 680 | 725 |

| Mean Fluorescence Intensity (MFI) | | | |
| --- | --- | --- | --- |
| CCL2 | | | |
| LPS (ng/ml) | N1 | N2 | N3 |
| 0 | 676 | 725 | 665 |
| 0.01 | 655 | 655 | 656 |
| 0.05 | 642 | 678 | 663 |
| 0.1 | 589 | 705 | 652 |
| 1 | 660 | 658 | 670 |
| 10 | 702 | 688 | 675 |
| 100 | 698 | 658 | 680 |
|  |  |  |  |
|  |  |  |  |
| Mean Fluorescence Intensity (MFI) | | | |
| CCL3 | | | |
| LPS (ng/ml) | N1 | N2 | N3 |
| 0 | 582 | 547 | 569 |
| 0.01 | 562 | 569 | 570 |
| 0.05 | 548 | 526 | 541 |
| 0.1 | 578 | 555 | 571 |
| 1 | 523 | 587 | 559 |
| 10 | 566 | 564 | 569 |
| 100 | 589 | 563 | 580 |
|  |  |  |  |
|  |  |  |  |
| Mean Fluorescence Intensity (MFI) | | | |
| CCL4 | | | |
| LPS (ng/ml) | N1 | N2 | N3 |
| 0 | 562 | 548 | 559 |
| 0.01 | 598 | 568 | 587 |
| 0.05 | 602 | 589 | 599 |
| 0.1 | 632 | 602 | 626 |
| 1 | 644 | 546 | 599 |
| 10 | 611 | 567 | 593 |
| 100 | 578 | 602 | 594 |
|  |  |  |  |
|  |  |  |  |
| Mean Fluorescence Intensity (MFI) | | | |
| CXCL10 | | | |
| LPS (ng/ml) | N1 | N2 | N3 |
| 0 | 679 | 647 | 668 |
| 0.01 | 691 | 620 | 661 |
| 0.05 | 707 | 618 | 668 |
| 0.1 | 670 | 656 | 668 |
| 1 | 704 | 620 | 667 |
| 10 | 702 | 652 | 682 |
| 100 | 658 | 658 | 663 |
