# Supplementary material for: Very low concentration of lipopolysaccharide can induce the production of various cytokines and chemokines in human primary monocytes
Source: BMC Res Notes. 2022 Feb 10;15:42. doi: 10.1186/s13104-022-05941-4 (PMC8832778; doi:10.1186/s13104-022-05941-4)
Supplement: Supplementary file 7 — Additional file 7: Table S3. Data analyzed form flow cytometric profiles of each subject (according to Fig. 3 in the paper): lipopolysaccharide induces the production of TNF-α and IL-6 in monocytes. PBMCs were stimulated with the indicated concentrations of LPS. The intracellular TNF-α and IL-6 were determined by flow cytometry. CD14+ monocyte population of the three individuals (as indicated) were gated and mean fluorescence intensity of the expression of the indicated cytokines are shown. [file 13104_2022_5941_MOESM7_ESM.docx]

| Mean Fluorescence Intensity (MFI) | | | |
| --- | --- | --- | --- |
| TNF-alpha | | | |
| LPS (ng/ml) | N1 | N2 | N3 |
| 0 | 637 | 458 | 558 |
| 0.000625 | 719 | 659 | 712 |
| 0.00125 | 796 | 708 | 790 |
| 0.0025 | 1039 | 1002 | 1121 |
| 0.005 | 1367 | 1125 | 1346 |
| 0.01 | 2316 | 2562 | 2563 |
| 0.05 | 8872 | 6687 | 7789 |
| 0.1 | 13461 | 10256 | 11858 |
| 1 | 13256 | 10587 | 12154 |
| 10 | 13568 | 11023 | 12295 |
| 100 | 13940 | 10985 | 12563 |

**Table S3. Data analyzed form flow cytometric profiles of each subject (According to figure 3 in the paper):**

**Lipopolysaccharide induces the production of TNF-α and IL-6 in monocytes.** PBMCs were stimulated with the indicated concentrations of LPS. The intracellular TNF-α and IL-6 were determined by flow cytometry. CD14+ monocyte population of the three individuals (as indicated) were gated and mean fluorescence intensity of the expression of the indicated cytokines are shown.

| Mean Fluorescence Intensity (MFI) | | | |
| --- | --- | --- | --- |
| IL-6 | | | |
| LPS (ng/ml) | N1 | N2 | N3 |
| 0 | 761 | 697 | 729 |
| 0.000625 | 882 | 907 | 900 |
| 0.00125 | 981 | 1008 | 1025 |
| 0.0025 | 1128 | 1378 | 1356 |
| 0.005 | 2655 | 3405 | 3458 |
| 0.01 | 4047 | 4350 | 4359 |
| 0.05 | 12560 | 4582 | 8788 |
| 0.1 | 15697 | 8956 | 12689 |
| 1 | 15589 | 9014 | 12798 |
| 10 | 15897 | 10258 | 12859 |
| 100 | 15982 | 10654 | 12956 |
